# A comprehensive review on isolated and non-isolated converter configuration and fast charging technology: For battery and plug in hybrid electric vehicle

**DOI:** 10.1016/j.heliyon.2023.e18808

**Published:** 2023-08-04

**Authors:** M.C. Annamalai, N. Amutha prabha

**Affiliations:** School of Electrical Engineering, Vellore Institute of Technology (VIT), Vellore, India

**Keywords:** Battery electric vehicle, Current ripples, DC-DC converter, Efficiency, Fast charging, Plug in hybrid electric vehicle, Power losses, Voltage ripples

## Abstract

Electric vehicle systems are a promising future transportation system because they play an important role in reducing atmospheric carbon emission and have become a focal point of research and development in the present era. The emerging fast charging technology has the ability to have refueling experiences comparable to gasoline cars. This article discusses existing electric vehicle charging infrastructure with a particular emphasis on rapid charging technologies, which would be needed to meet current and potential EV refueling requirements. Various dc-dc converter topologies for battery electric and plug-in hybrid vehicles are compared and contrasted in this article in terms of performance, output power, current ripples, voltage ripples, conduction loss, recovery loss, switching frequency loss, reliability, durability, and cost. The architecture, benefits, and drawbacks of AC-DC and DC-DC converter topologies for rapid charging stations are also discussed in this article. Furthermore, this study addresses the crucial problems and difficulties associated with electric vehicle converters for direct current rapid charging. Eventually, technical and relevant contributions are provided for an electric vehicle system development.

## Nomenclature

AC: Alternating current
AFID: Alternative Fuels Infrastructure Directive
AFIR: Automotive Future Impact Report
BESS: Battery Energy storage system.
BSS: Battery storage system
BMS: Battery management system
CO_2_: Carbon dioxide
CCS: Combined Charging System
CAN: Controller Area Network
CC: CUK converter
CIBC: Coupled inductor and Bidirectional converter
CC: Constant current charging
CV: Constant voltage charging
CC-CV: Constant current -Constant voltage charging
DC: Direct current
DCFC: DC fast charger
DG: Diesel generator
DSM: Demand side management
DR: Dynamic rating
EV: Electric Vehicles
ESS: Energy storage system
EENS: Energy-not-served index
EMI: Electromagnetic interference
FC: Fast charger
GaN: Gallium nitride
GNEP: Generalized Nash equilibrium problem
GPS-BD: Global positioning system -Beidou navigation positioning module
GB/T: GuoBiao/Tuijian
HD: Heavy duty
HV: High voltage
HBGM: Half bridge gallium nitride modules
ICT: Information and communications technology
IEC: International Electrotechnical Commission
LQR: Linear quadratic regulator
LV: Low voltage
LFP: Lithium iron phosphate
MPAS: *Model for Prediction Across Scales*
MDBIC: Multidevice interleaved bi-directional converter
MDIBC: Multidevice interleaved bi-directional converter
MSCC: Multistep constant current charging
MV: Medium voltage
NPC: Neutral point clamped converter
NTO: Network topology optimization
NMC: Nickel-manganese-cobalt
OEM: Original equipment manufacturers
PBEV: Plug in Battery Electric Vehicles
PEVs: Plug-in electric vehicles
PCB: Printed Circuit board
PFC: Power factor correction
PWM: Pulse with modulation
PC: Pulse charging
QZBC: Quasi z-source with Bidirectional converters
RE: Renewable energy
RES: Renewable energy system
RTTR: Real time thermal rating
RTN: Reliability test network
SPN: Secondary pore network
SAE: Society of Automotive Engineers
Sic MOSFET: Silicon carbide metal oxide semi-conductor field effect transistor.
SCBC: Switched capacitor bi-directional converter
TRL: Technology Readiness Level
TEN-T: Trans-European Transport Network
UFC: Ultra fast charger
WPT: Wireless power transfer
XFS: Extreme fast charging
ZVS: Zero voltage switches
ZCS: Zero current switch
ZVSC: Zero - Voltage switching converter

## 1 Introduction

Electric vehicles (EVs) have grown in popularity in recent decades as a result of their superior performance and efficiency. With greenhouse gas pollution and the global warming and fossil fuel decline, electric vehicles have generally been recognized as replacement for global environmental problems and CO_2_ emissions in the automotive industry [1]. Power electronic technologies provide great dependability and efficiency in the conversion of renewable energy, as well as assisting in the reduction of harmful global emissions [2]. The calculated greenhouse gas mitigation in 2030 is 40–215 Mt CO_2_ and in 2050 is around 340–1380 Mt CO_2_ in PBEV. It arises the decarbonization in the transport sector [3]. A portable emission measuring system measures carbon dioxide emission. CO_2_MPAS methodology used to calculate the CO_2_ emission accurately [4]. Low electricity prices and high oil prices drive electric vehicles' diffusion rate to 60–70%. Manufacturers' production subsidies have a more significant impact on electric vehicles than consumer purchase subsidies [5]. Biobatteries have the potential to be a promising green battery technology for the future, with lower environmental impact than current metal lithium batteries, both for compact systems and the automobile industry [6]. Electric vehicles employing Lithium-ion batteries face difficulties owing to different internal and external factors, in projecting precise health and remaining functional life [7]. The energy supply and power control are the serious problems in electric vehicles with an ultracapacitor and battery hybrid power storage system for proper management of the power distribution between the available drive systems [8]. A distributed power generation and electric charging approach was introduced to minimize the operational costs of distribution systems, including uncertainties on DG output power and the spontaneous charging power to electric vehicles (EV) [9]. Analysis relies on generalized Nash equilibrium (GNEP) for controlling the charging of plug-in electric vehicles (PEVs) in a delivery grid [10]. Conception of a bidirectional linearized dc-dc converter that is used in energy consumption and recovery units, especially in compact hybrid electric vehicles [11]. The Bidirectional dc/dc converter integrates primary energy storage, secondary energy storage, and a dc-bus with changing voltage ratios in a hybrid electric vehicle system. Two modes operate the bidirectional power control: with dc, a low voltage dual power supply and a high voltage regenerative energy [12].

With the help of a neutral auxiliary circuit, a ZVS full-bridge dc-dc converter for traction battery charging is made up of ac switches that turn on at zero voltage throughout the complete load range. Traditional full-bridge dc-dc converters use primary-side diode clamping circuits to prevent voltage spikes on the secondary rectifiers [13]. Quadratic as well as switched capacitor configurations are used in electric vehicle applications. A bi-directional dc-dc converter ensures high voltage conversion levels, minimizes voltage stress on semiconductor devices, and maintains a constant potential difference between higher and lower voltage ports and then a constant current also at low voltage terminal. Synchronous rectification is indeed a technique for improving the performance of a converter [14]. After removing the resonant peak, the design approach of the Filter (Wideband) for the HV input port and the PCB-level filter design technique solved the problem that an CISPR25-2016 requirement is exceeded by vehicle converters for high-voltage/low-voltage dc-dc interference voltage [15]. For automotive Electric vehicle a buck boost half-bridge with a double input three levels play an essential role by converting energy from rechargeable batteries and fuel cells to the accelerator and ultra-capacitor during the braking process. The converter can provide a high-power density and also a reliable energy conversion because of unregulated low voltage in the battery and fuel cell [16]. This charging system is termed to substitute the alternator of the car and also to ensure efficient braking contact for driver even though regenerative braking energy is recovered in the main HV battery to be stored. Furthermore, the total efficiency of a charging dc-dc circuit is more than the car alternator and therefore could be easily improved [17]. Charging architecture is classified in the single-way and two-way power flow direction into an on-board and off-board charger. The interconnection problem in unidirectional charging may restrict the hardware requirement, but the two-way charge supports battery energy storage back to the grid [18]. It can provide a fueling experience in comparison with gasoline vehicles, a technology for high-speed charging used to resize the vehicles infrastructure [19]. The revolution towards sustainable energy gives flexibility to DC fast charging. In DC, maintenance costs and costly grid reinforcement are offset by quick charging, which reduces the need for additional energy storage [20]. The extent of permanent storage systems will increase profitability by reducing the costs of grid connections in city and highway charging stations [21]. The infrastructure of powerful charging stations that can mitigate the gasoline stations is being developed by fast-growing electric vehicles. The charger in the EV battery can be refilled easily by means of a simple charger offboard DC [22].

The electric vehicle’s market share is to be increased by reducing the battery’s charging time, but it is limited by poor electrolyte transport. The electrochemical modal of 2D Physics with SPN in either one or two electrodes used to boost the electrolyte transport [23]. The virtual sync algorithm manages the bandwidth, harmonic reduction and compensate for the reactive power in the power grid, mitigating the impact on electric cars of the ultra-fast loading stations [24]. The smart grid model, including intelligent computers, ICT and energy storage networks, is combined with power station systems for electric vehicles [25]. A full a control strategy for an electric drive system built into the wheels has to increase lateral vehicle stability when network-induced time delays are considered. A concern with network-induced time delays in a linear quadratic regulator (LQR). Has been devised to reduce reference state monitoring errors and control effort [26]. A global positioning system and Beidou navigation positioning module (GPS-BD) as well as a low-cost initial measurement device are employed in a multi sensor fusion-based length vehicle speed estimate methodology for four-wheel autonomous electric cars [27]. New Electric Vehicles have insufficiently accurate range estimators. To reduce this issue, a specific power-based Electric drive energy usage model is required to determine an exact range estimate [28]. In order to have the same driving rating and user acceptability as current petrol cars, Batteries only need 666 kg^−1^ at cell level. The most advanced ultra-capacitors focused on nanotechnology would certainly not match the battery technology’s energy capability [29]. Multi-energy sources are used in Bidirectional converter and exchanges energy. It will be operated in stepdown and step-up mode. In breaking mode also, the battery will be charged. It is mostly used in electric vehicle [30].

According to the literature review, most studies have addressed the general theory underpinning the evolution of the charging infrastructure. Regrettably, there needs to be more clear advanced literature describing charging architecture, topologies, methodologies, and suggestions for the future. Thus, the following additions are made by this work relative to the current literature: This article seeks to give a detailed and up-to-date assessment of PEV charging designs, focusing on conductive and inductive charging techniques. The primary objective of this study is to assess the present needs, recent breakthroughs, and complex challenges associated with power electronics converters to propose prospective enhancements to the charging ways of electric cars. Isolated and non-isolated topologies utilizing soft switching methodologies are characterized and thoroughly studied based on their respective constraint assessments. This research focuses on innovative power conversion architectures utilizing modular multi-level converters that facilitate the optimal integration of plug-in electric vehicles (PEVs) with the primary power grid through efficiently utilizing distributed energy storage systems and renewable energy sources. The presented topologies have been researched and assessed in terms of scalability, efficiency, and peak charging power, with their impact on the primary grid’s power quality taken into account. This article’s perspective covers the many types of charging methods for EV batteries, identifies various techniques to maximize energy storage systems, examines a hybrid energy storage system, and investigates the benefits of a swappable energy storage system. The research gap identified in the existing research that can be filled by additional investigation in the future.

The layout of this review paper is split into six parts. Section II parallels this presentation by presenting a charging infrastructure for electric car applications. A summary of dc-dc converters used in quick charging technologies is presented in section III. Segment IV deals with relevant topologies of dc-dc converters in order to electric battery cars, electric hybrid vehicle plug-in along with rapid-charge converter topologies. Section V discusses a critical comparison of charging methods and probable future research approaches in designing and developing new rapid charging technologies to meet commercial and social needs. Finally, RE-based freestanding hybrid EV charging stations' ESS designs are described in Section VI. Section VII investigates the recent development of FC, its architecture, and its infrastructures. Section VIII of this paper provides an overview of the charging technology used for heavy-duty electric vehicles in the European Union (EU).

## 2 Infrastructure for electric vehicle charging

### 2.1 Charging standards

Due to major fluctuations, EV charging standards can vary according to the parameters of the global Low Voltage Grid, such as voltage and frequency either be locally or nationally standardized, with global errors [31].

The IEC standards are also found in European countries. The SAE principles are mostly used in the United States of America. China is the primary consumer of the GB/T specifications [32]. The Automotive Engineers' Company (SAE) of North America defines conductive methods for charging EVs in the standard SAE J1772 [33]. GB/T 20234.2 and GB/T 20234.3 are detailed specifications for AC and DC conductor charging connectors [34]. The wireless power transfer (WPT) interface transmission protocol includes the IEC 61980- 2 specification for electric vehicles (EVs). It specifies the communication standards for EVs as well as charging infrastructure. A main list of these standards is given in Table 1 [35].

**Table 1:** Electric Vehicle charging Standards.

STANDARD	IEC [35]	SAE [33]	GB/T [32]
Connector	62196-1	J1772	20234-1
	62196-2		20234-2
	62196-3		20234-3
Communication	61850	J2293-2	27930
	61980-2	J2836	
	61980-3	J2847	
Topology	61439-5	J2953	18487-1
	61851-1		29781
	61851-21		33594
	61851-22		
Safety	60364-7	J1766	18384-1
	60529		18384-3
	61140	J2894-2	37295
	62040		

### 2.2 Charging modes

A variety of charging modes have recently been introduced to address the basic needs of electric vehicles in various contexts. The charging modes defined by the SAE J1772, IEC 61851-1, and GB/T 18487-1 standards are commonly used around the world [36]. The most popular AC slow loading mode is AC Level 1, which can be easily accomplished using the inboard power converter from a household socket. Normally, the Model 1 takes several hours to charge in this situation [37]. A safety mechanism integrated into the cable connecting the EV to the connector is a possible solution to this issue when charging from a domestic socket outlet. When operated through this charging mode, known as Mode 2 (AC Level 2), can reach a power demand of up to 19.2 kW by using a 3∅ voltage AC source [38]. AC quick charging is known as Mode 3. Special power units with control pilot function are needed as EVs are linked to the power grid to resolve changes in AC voltage and frequency values between regions and meet the need for increased charging capacity [38]. With a power level of 400 kW, Mode 4, when used in conjunction with a DC offboard charging unit, is a viable option. Tesla also specifies the charging mode specifications. According to the most widely used guidelines, Table 2 lists conductive charging device modes [38].

**Table 2:** Charging modes of conductive charging devices [[36], [37], [38]].

AC/DC	Levels for charger	Maximum voltage	Maximum current	Power Level	Estimated charging time
STANDARDS OF SAE					
AC	Level I (On-board)	120 V (US), 230 V (EU)	20 A 12 A	1.9 KW 1.4 KW	7–17 h
AC	Level II (On-board)	240 V (US), 400 V (EU)	32 A 80 A	8 KW 19.2 KW	0.4– – 7 h
DC	Level I (Off-board)	(200–450) V	80 A	40 KW	0.4–1.2 h
DC	Level II (Off-board)	(200–600) V	200 A	90 KW	0.2–0.4 h
DC	Level III (Off-board)	(200–600) V	400 A	240 KW	0.1–0.2 h
**STANDARDS OF IEC**					
AC	Level I (On-board)	1∅, 250 V	16 A	4–7.5 KW	7 h
		3∅, 480 V,	16 A		
AC	Level II (On-board)	1∅, 250 V	32 A	8–15 KW	3 h
		3∅, 480 V	32 A		
AC	Level III (On-board)	480 V	250 A	60–120 KW	30 min
DC	Level IV (Off-board)	500 V	400 A	1000–2000 KW	<0.1 h
**STANDARDS OF GB/T STANDARDS**					
AC	Level 1	1∅, 250 V	8 A	27.7 KW	1.5 h–8 h
AC	Level II	1∅, 250 V	16 A		
AC	Level III	1∅, 250 V	32 A		
		3∅, 440 V	63 A		
DC	Level IV	750 V, 1000 V	250 A	250 KW	<0.1 h

### 2.3 Connector for charging

**Fig. 1:**
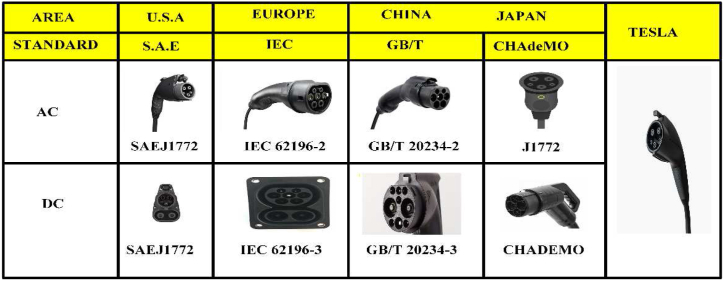
Charging outlets of EV_s_ connector.

Various charger plug types configurations are used depending on the charging conditions and modes of various countries. Mennekes connectors of types 1 and 2, which are based on SAE J1772 and IEC 62196-2, are frequently used for AC charging operations in the United States and Europe. Often level 1 and level 2 connectors are mostly used for chargers [39]. Combined Charging System (CCS) connectors use both the Type 1 and Type 2 charging pins and two most electric vehicle users in the world use the GB/T234 socket [40]. The CHAdeMO connector, made by Tepco, became the official Japanese DC charger standard. There are two main pins for sharing of power and one for touch. The communication protocols CAN is used for GB/T and CHAdeMO, while PLC is used by the others. In actuality, Tesla offers a proprietary cable which can support AC and DC in the charging stations of Tesla only. The proprietors will also obtain an adaptor for the Tesla charger that enables their cars use of category 1 connector charging points. Fig. 1 summarizes the key charging outlets for electric car connectors [41].

### 2.4 Charging system

**Fig. 2:**
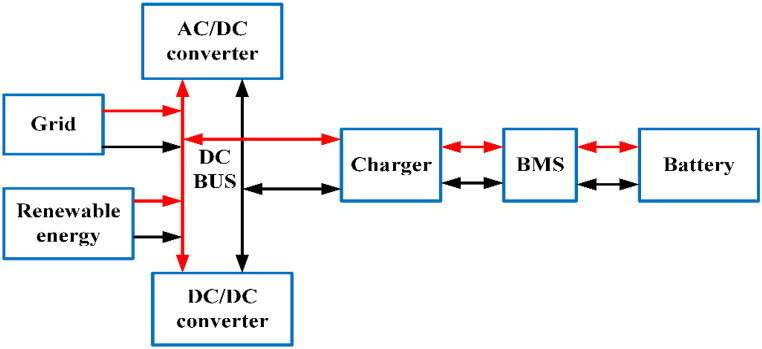
Electric Vehicle Charging system.

EV charging systems typically have several power outlets, inverters/converters, and an internal battery [42]. Fig. 2 depicts a conventional green energy-powered grid-connected charging device. Renewable technology can be used to charge EVs and excess power is sent converters/Inverters to grid. Combine various sources of energy to power electric vehicles with a common power source. The battery management system (BMS) controls battery resources and communicates with the battery [43]. Table 3 shows commercially available of OFF-board EV battery charger and Table 4 shows specification of commercially available EV battery charger.

**Table 3:** Specification of OFF-board EV battery charger [44].

Manufacturer	ABB	TRITIUM	PHIHONG	TESLA	EVTEC	ABB
**Model**	Tera 53	Veefil - RT	Integrated Type	Supercharger	Espresso & charge	Terra HP
**Power**	50 KW	50 KW	120 KW	135 KW	150 KW	350 KW
**Input voltage**	480Vac	380–480 Vac 600–900 Vac	380 Vac–480 Vac	380–480 Vac	400 Vac	400 Vac
**Output voltage**	200–500 V	200–750 V	200–750 V	50–410 V	170–500 V	150–920 V
**Output current**	120 A	125 A	240 A	330 A	300 A	375 A
**Connector**	CCS Type-1 CHAdeMO 1.0	CCS Type 1&2 CHAdeMO 1.0	GB/T	Supercharger	SAE combo-1 CHAdeMO 1.0	SAE combo-1 CHAdeMO 1.0

**Table 4:** Specification of commercial EV battery charger [44].

EV Model	Nissan Leaf	Renault Zoe R135	BMW X3	VW ID.4 Pro S	Hyundai IONIQ 5	Chevrolet Bolt	Tesla Model Y	JAC IEV7S/E	Chery eQ
**Year**	2021	2020	2021					2019	2020
**Region**	Europe					US		China/Japan	
**Motor power (KW)**	110	100	125	150	160	150	201	50	30
**Battery capacity (KWh)**	40	54.6	43	82	73	66	75	24	22
**Charging time**	3 h 22 min	2 h 22 min	3 h 15 min	7 h 30 min	6 h 9 min	10 h	7 h 30 min	2 h 26 min	2 h 14 min
**OBC Rating (KW)**	11	43	11	11	11	6.6	11/22	11	11

## 3 Converter topologies for fast charging

### 3.1 AC-DC converter for quick charging

**Fig. 3:**
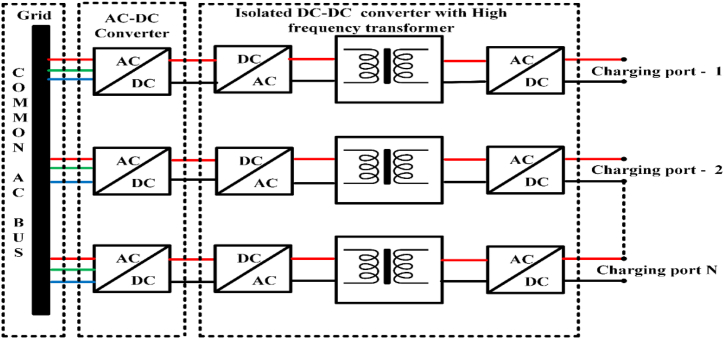
AC bus Architecture.

The AC bus architecture as shown in Fig. 3, the Fast-Charging Architectural design includes high-frequency transformers stages for AC-DC conversion and, while the dc bus design stage low-frequency transformer includes a specific AC-DC conversion stage. According to the SAEJ1772 standard, 600 v and 550 A of dc voltage in fast charging to charge the electric car in less than 10 min, the electric vehicle should be charged, and a quick charger should be put outside the vehicle [45].

#### 3.1.1 Bridgeless 3∅ boost converter

**Fig. 4:**
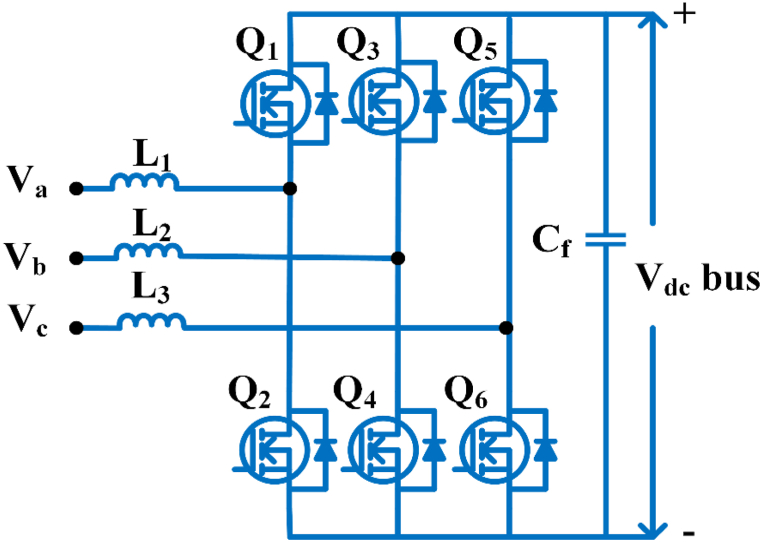
Three phase boost converters without a bridge.

The 3∅ boost ac/dc full-bridge topology for the power conversion of higher power factor values is corrected as shown in Fig. 4. Any changes in load and phase resistances alter the sliding mode regulation for the optimal output voltage and any uncertainties, resulting in a power factor value close to unity [46]. This topology explores the application of hysteresis regulation with frequency constant to a 3∅ boost converter. When converter output voltage exceeds the maximum phase neutral input voltage by three times, this technique is employed [47].

#### 3.1.2 Vienna rectifiers, three phases

The Vienna rectifier is a 3∅, three-level rectifier used to increase power factor, decrease the harmonic current, and reduce reactive power pollution in a dc charging pile. The current hysteresis regulation is used to control the Vienna rectification mechanism swiftly and stably [48]. The topology employs virtual synchronous motor power on the Vienna rectifier to increase flexibility of the grid to large-scale electric vehicle access. Electric vehicle chargers with periodic load swapping maintains the output voltage stability and low total harmonic distortion of current, allowing the power device to respond to voltage and frequency fluctuations and increase inertia damping at the interface. It offers the advantages of a high-power factor and low switch voltage tension, a high-power density, and a steady output voltage, and it is increasingly being used for the front-end stage of the AC/DC component of electric vehicle chargers [49]. The finite set model predictive control algorithm for Vienna rectifier for electric vehicle charger is used to control input current distortions without requiring the importance of converter idealities and their variants [50].

**Fig. 5:**
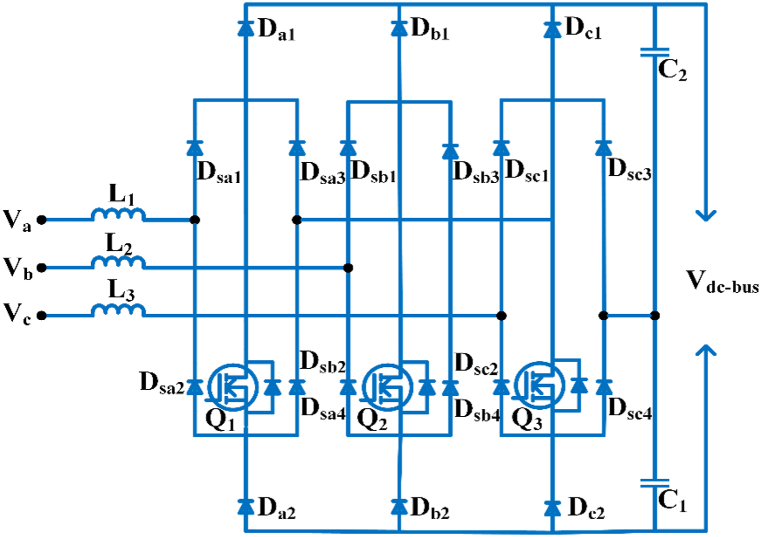
Three-phase Vienna rectifier.

The key switches Q_1_, Q_2_, and Q_3_ are all switched off, so the minimum voltage in each capacitor filter C_1_ and C_2_ is the input’s maximum line-to-line voltage. Therefore, the converter’s minimal boost voltage requirement is double the line-to-line voltage as seen in Fig. 5 [51].

#### 3.1.3 Three phases three-level neutral point clamped converter

**Fig. 6:**
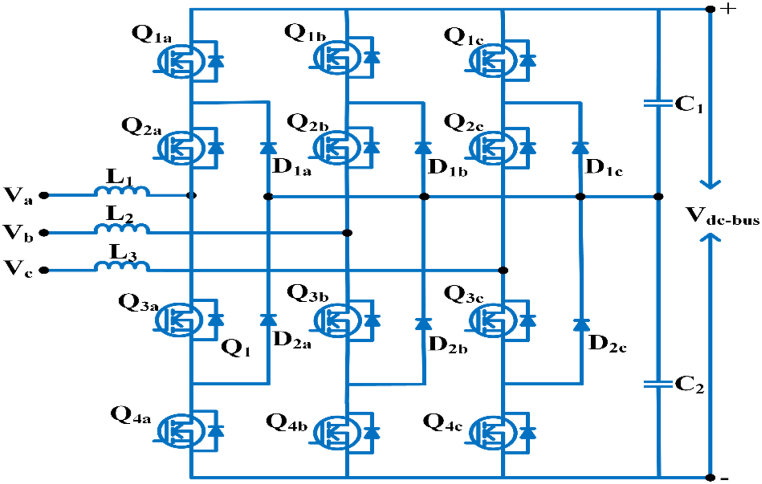
Three phase three-level NPC converter.

The central converter topology is a neutral point clamp converter, and a novel balance strategy is used to increase the operating range of the power converter in electric vehicles [52]. A three-phase dual output neutral point clamped three-level inverter topology produces two groups of alternating current and voltage outputs with adjustable frequency and amplitude. It resolves the neutral point voltage balance and acknowledges circuit modulation [53]. As seen in Fig. 6, a 3∅ three-level neutral point converter provides several options for linking input sources and delivering a five-level output voltage. As a result, the dv/dt rating is lower, and the filtering requirement is reduced. The negative and positive dc buses have a power imbalance that causes grid-side current instability [54]. Table 5 shows the comparative analysis of AC/DC topologies and Table 6 shows specification of AC-DC converter for quick charging.

**Table 5:** Comparison of AC/DC Topologies [46,48,52].

	2-Level	3-Level NPC	3-Level Vienna
THD of output current	High	Very Low	Very low
Peak voltage stress on active and passive devices	High	Low	Low
Switching loss	High	Low	Mid
Power density	Low	Higher	High
Conduction loss	Low loss	Very- High loss	High loss
Efficiency	Low ɳ	Very- high ɳ	High ɳ
Cost	Low cost	High cost	Moderate cost
Control	Easy	Moderate	Moderate
Input inductor size	Large	Low	Low
Thermal management	Vast	Challenging due to unsymmetrical loss distribution	Mild
Bidirectional	Yes	Yes	No

**Table 6:** Specification of AC-DC converter for quick charging.

Topology	Power (KW)	Frequency (KHz)	Output voltage (V)	Number of Switches/Diodes	THD	Efficiency
Bridgeless 3 phase boost converter [47]	1.5	200	400	6/8	<1.77%	98.2
Three phase Vienna Rectifier [[49], [50], [51]]	1.8	50	700	6/6	3.5%	98.8
Three phase three level neutral point clamped converter [53,54]	30	10	858	12/6	2%	97

### 3.2 DC-DC converter for quick charging

**Fig. 7:**
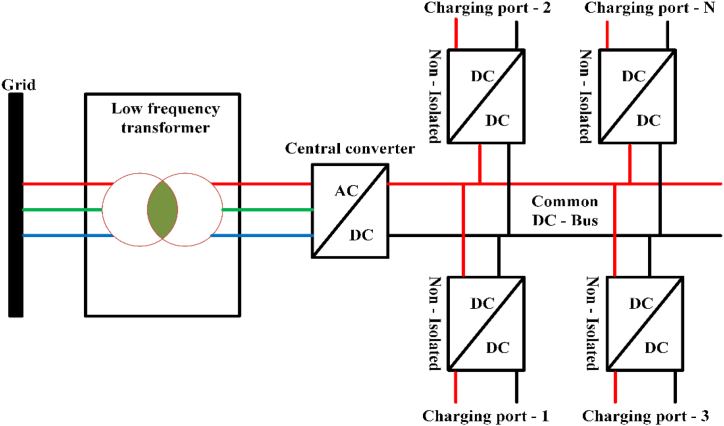
DC bus charging station.

The dc bus charging station as shown in Fig. 7. There are three steps involved in the charger. The rectifier power factor is controlled, and the DC-DC converter receives a steady dc voltage. While the DC-DC converter stage can be used to regulate the charging current for improved response [55]. Unipolar and bipolar dc converters are utilized for ac-dc/dc-dc conversion in electric vehicle fast charging application [56].

#### 3.2.1 Multiple interleaved buck converter

The Multiple interleave buck converter has low voltage tension and a high step-down conversion ratio, and it is most widely used in wherever requiring a lesser voltage in output by giving a high input. The switches are linked together with connecting capacitors; it works under less than 50% of duty cycle with high frequency [57].

**Fig. 8:**
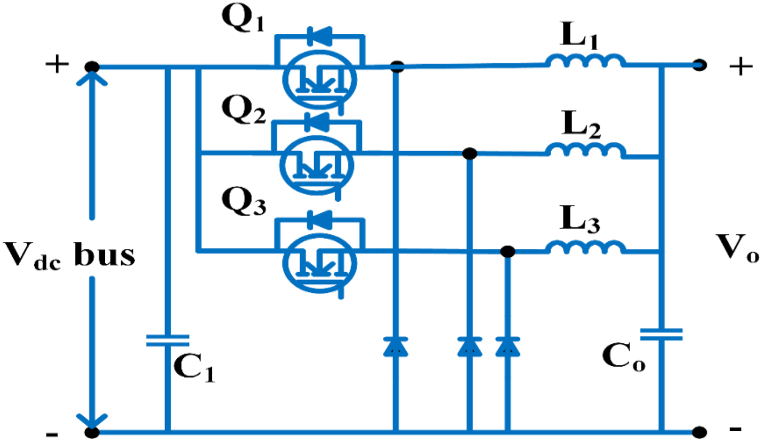
LLC buck converter with multiple interleaved stages.

A buck converter with a single phase is used to power the batteries. It has a high inductor value, which raises the cost and scale, but this is mitigated by using a multiphase interleaved buck converter, as seen in Fig. 8. The converter distributes current among the multiphase modules and reduces the size of the inductor. This converter is not suitable for a device where a front transformer is present at high frequency harmonics [58].

#### 3.2.2 Full bridge LLC resonant converter

**Fig. 9:**
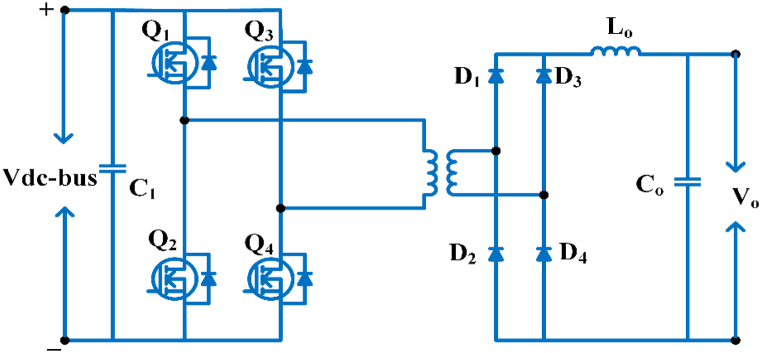
Full bridge LLC Resonant converter.

The topology is used for electric car charging. High efficiency, volume, electrically isolation, minimal electromagnetic interference, low voltage tension, a broad output voltage range, and a high operating frequency are all advantages of this converter [59]. The bridge resonant converter for dc-dc conversion as indicated in Fig. 9, for fast charging. It achieves zero voltage switching, with no rectifier diode oscillation voltage and no reverse recuperation current [60]. The major downside is to control the battery’s output voltage, this topology necessitates the use of a broad range of switching frequencies this complicates the transformer and filter design, and when the battery voltage is down, the converter’s operation becomes less efficient [61].

#### 3.2.3 Full bridge phase shift converter

**Fig. 10:**
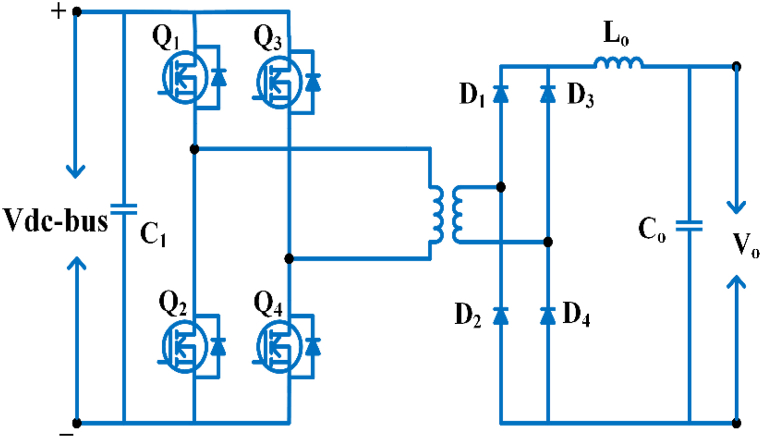
Bridge phase shift converter.

A full-bridge phase shift converter used in control applications as shown in Fig. 10. These converter feature are, a fast control mechanism, low current stress and soft switching for the primary switches. This converter has a number of drawbacks, including excessive flowing of current during the freewheeling and excessive voltage stress on the bridge rectifier [62]. Table 7 shows the comparison of dc/dc topologies and Table 8 shows the specification of dc/dc topologies.

**Table 7:** Comparison of dc/dc topologies [59,62].

	Full bridge - LLC resonant converter	Full bridge-phase shift converter
Peak device stress on primary and secondary side	High	Mid low
Transformer KVA rating	High	Medium
Power output to transformer KVA rating	Low	Medium
Input and output capacitor RMS currents	High	Medium
Operation	Unidirectional	Unidirectional
Conduction losses	High	Medium
Control complexity	Moderate	Very simple
Wide battery voltage, fixed bus voltage	No, needs additional DC/DC stage	Yes (with reduced efficiency)
Paralleling modules	Intensive	Easy
Switching frequency	Fixed/High/(Si/SIC)	High
switching loss when Turn ON	ZVS	ZVS
switching loss when Turn OFF	Low (ZCS)	High
Total losses	Low	Higher

**Table 8:** Specification of dc/dc topologies.

Topology	Power	Freq (KHz)	Output voltage(V)	No. of Switches/Diodes	Efficiency
Phase shifted Full Bridge converter [62]	1.2	100	209–350	4/4	95%
LLC Full bridge resonant converter [60,61]	3.3	83.33–220	250–450	4/4	98.2%
Interleaved buck converter [58]	1	16-32	350	4/4	98.5

## 4 Converter classification of battery and plug-in hybrid electric vehicle

**Fig. 11:**
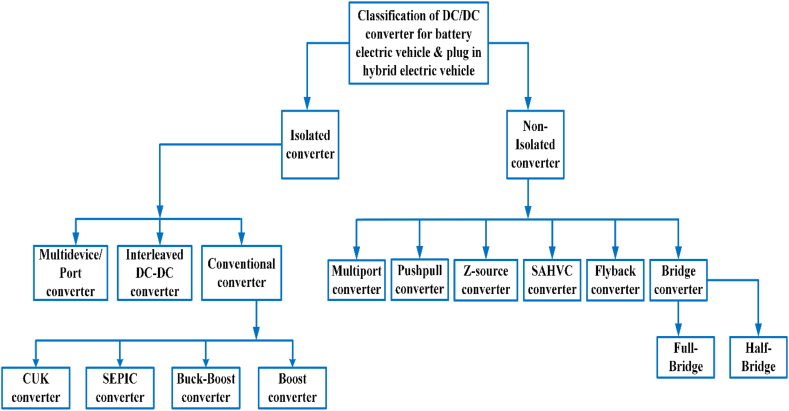
BEVS & PHEVS converter classification.

As seen in Fig. 11, electric vehicle converters are Classified in two different types: non-isolated and isolated. In electric vehicle for medium to high power non-isolated converters are utilized, while low and medium power isolated converters for use in hybrid cars [63].

### 4.1 Non-isolated dc-dc electric vehicle converter

#### 4.1.1 Cuk converter

**Fig. 12:**
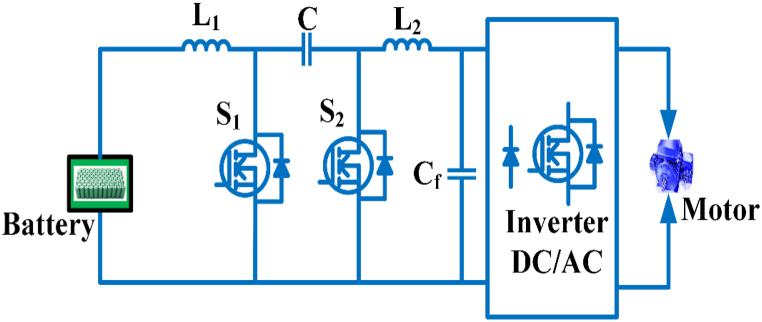
CUK converter.

The output voltage is inverted and lesser or higher than input voltage is cuk converter. The topology for an Electric vehicle is designed using two switches, two inductors, and two capacitors, as shown in Fig. 12 A capacitor is the primary energy storage portion, where current flows continuously. The coils will share a common magnetic core, reducing ripple and increasing efficiency. The energy flow is bi-directional by using a diode and a switch. The inductors L_1_ and L_2_ transform the energy from the battery storage system to the electric motor. It can operate current operating modes both continuously and discontinuously [64]. The voltage gain can be written as

(1) $$\frac{Vo}{Vi}=\frac{-D}{1-D}$$

The cuk converter combines a buck and a boost converter with zero ripple. It consists of a single switching system and a shared capacitor. At the input, the DC voltage is transferred with reversed polarity to DC voltage on the output. The cuk converter with a single switched inductor in an Electric vehicle offers high step-up or step-down gain, less current ripple, and reduced component. The cuk converter makes the constant input current and the output stress to be more or less than the input stress compared to Buck, Boost and Buck-Boot converter [65].

For high power factor adjustment, the Bridgeless cuk converter is used in electric vehicle battery charges. The charger efficiency is increased, and conduction loss is reduced by operating over one switching cycle, and thereby added advantage is unwanted capacitor coupling is removed [66].

The topology of an extra winding, auxiliary diode, and auxiliary inductor are used in discontinuous capacitor voltage mode of a soft-switching. It attains soft-switching operation without any active switches, and it reduces switching losses and also recycles the leakage inductance energy [67]. In Bridgeless PFC cuk converter, when operating in discontinuous conduction mode the efficiency is satisfactorily improved in the Electric Vehicle charger and battery current is regulated by using constant current and constant voltage methods. It eliminates the extra inverse amplifier to change the polarity conversion. The advantage is a reduction of sensor and zero current switching [68].

#### 4.1.2 Bidirectional converter and coupled inductor

**Fig. 13:**
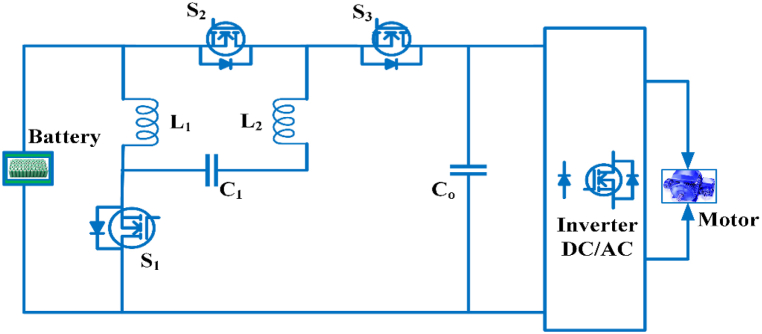
Coupled inductors with Bi-directional converter.

In comparison to other converters, the coupled inductor with a bidirectional converter for a single magnetic core electric vehicle application demands a lower volume of size as shown in Fig. 13 [69]. The voltage gain can be written as:

(2) $$\frac{V_{o}}{v_{i}}=\frac{2+n-D}{1-D}$$

The coupled inductor and voltage multiplier are combined to increase the gain of voltage. The remained stored energy is recovered by applying passive clamps, and efficiency is improved. The high step-up continuous input current makes it suitable for rapid charging of EV_s_ application [70]. Across the switches, the voltage stress is lower in the coupled inductor bidirectional converter. On the low voltage side, an interleaved technique achieves low current ripple, while phase shift angle modulation regulates the output voltage [71]. For high-power electrical applications, the interleaved boost converter’s coupled inductor is used to increase its efficiency and less electromagnetic emission. The Electrical performance is improved by connecting the inductor in the front end, which is magnetically coupled. The DC flux level in the core is negligible, and the ripple current in the inductor is less by providing an inverse coupling than directly coupled configuration [72].

In electric vehicle applications, the coupled inductor is primarily utilized for high voltage gain and low switch voltage tension and also alleviating output diode’s reverse recovery issues [73]. it is tested using the buck and boost modes of activity in electric vehicles, a bidirectional coupling inductor DC/DC converter is used to control the DC voltage [74]. In an electric vehicle application, the topology of a DC/DC bidirectional converter with inductor coupled conducts bi-directional flow of current between input source and 3∅ inverter [75].

#### 4.1.3 Bidirectional converters with quasi z-source

**Fig. 14:**
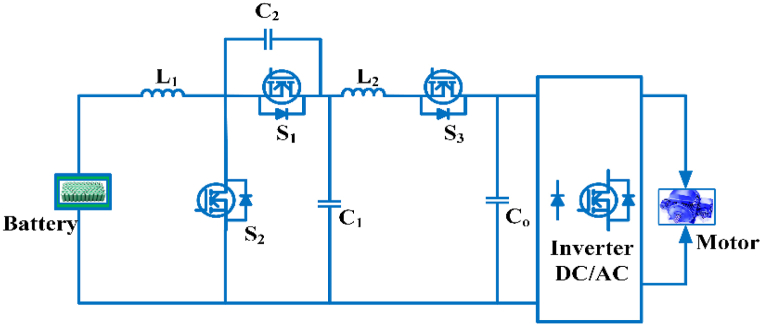
Bidirectional converter with quasi-z source.

The Bidirectional converters with quasi Z-source consists of three switches S_1,_ S_2,_ S_3_, three inductors, and three capacitors as shown in Fig. 14. The converter will work in either step-up or step-down mode, allowing power to flow between the low and high voltage sides. S1 is the primary control transfer in step-up mode; it is switched ON, and S_2_ and S_3_ are synchronous rectifiers; they are switched OFF. Power is passed from S_1_ to C_2_ and L_2_ after the capacitor C_1_ is discharged. The duty cycle is taken as d_1 =_ 1−d_2 =_ 1−d_3 =_ d_boost._ The voltage gain M_boost_ and duty cycle d_boost_ may be expressed as in continuous conduction mode

(3) $$M_{boost}=\frac{1+d_{boost}}{1-d_{boost}}$$

In step-down mode S_2_ and S_3_ operate as the main power switches, it is switched ON and S_1_ is the synchronous rectifiers is switched OFF. C_1_ is discharged from S_2_, while L_1_, L_2_, and C_2_ are charged from S_3_. The duty cycle is taken as d_2_ = d_3_ = 1−d_1_ = d_buck_ [76]. M_buck_ voltage gain and duty period d_buck_ in continuous conduction mode can be written as

(4) $$M_{buck}=\frac{d_{buck}}{2-d_{buck}}$$

Electric cars use DC-DC converters with a single switched capacitor that are quasi-Z-source. It provides a high gain in the output voltage, low output voltage stress across the diodes, less current ripples in the switches, and increased performance [77]. The switched Quasi Z-source inverter uses a switched impedance network, provides an excellent boosting capacity, low voltage stress through switches and capacitors, low power consumption and a continuous input current suitable for electric vehicle applications [78]. The dc-dc framework that has been built in extension of quasi-Z-source converters to n levels. It provides increased efficiency due to the increased voltage gain. In a traditional quasi-Z-source converter, if any part fails, the whole structure is disrupted. In this converter, the fault management system will isolate the stage and transfer power continuously [79]. In a hybrid energy system for an electric car, a switching quasi-Z-source dc-dc converter is used. Three simple control switches comprise the structure, and it benefits includes a considerably wider voltage gain bandwidth for an absolute common ground in step-up and step-down mode. The static and dynamic outputs are appropriate, and it can connect to a low voltage battery pack and a high voltage direct current bus in an electric car driven by a hybrid energy system [80]. A bidirectional inverter with a quasi-Z source is intended for use in a battery storage system, and various controllers are used to optimize the battery current stress. Time domain and frequency domain controllers are used to regulate power [81].

#### 4.1.4 Multidevice interleaved bi-directional converter

**Fig. 15:**
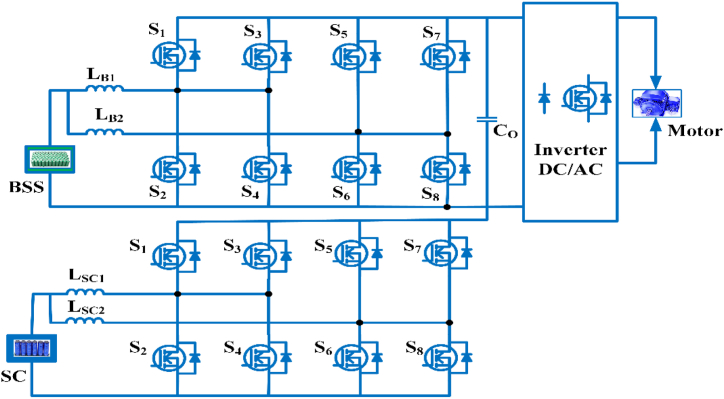
Multi-device Interleaved Bi-directional converter.

The Multidevice interleaved bi-directional converter configuration consists of two power sources, a battery storage system and switched capacitor as shown in Fig. 15. It makes use of interleaving techniques and drives multiple power stages in parallel to the gate signals, which were modified by 3600/n, where n is the step count. Switching frequency that is effective and is proportional to step count. Each switching system operates at the same frequency, but with an 180° phase difference. The current is splintering. Depending on the duty ratio, the switching sequences of each phases can overlap [82]. The duty cycle of MDIBC may be stated as

(5) $$D=\frac{1}{N}\left(1-\frac{V_{i}}{V_{o}}\right)$$

Bi-directional converter in three phases is popularly increase in Electric vehicles because it provides better performance and ensures output current ripple is minimum, high efficiency, and cost-effective [83]. To further maximize the output of the power train, the optimized power electronic interfaces for battery electric vehicles it often handles power flow for each operating mode. This converter can help to minimize the active and passive elements size, ripples of voltage and current and also increasing the device reliability of battery-electric vehicles [84]. The converter is primarily suitable for a hybrid energy source in electric vehicle load. Load power is flexibly distributed between input sources. Voltage harmonics, torque ripples in motors are reduced by using a multilevel inverter in Electric vehicles. Two distinct operations are used to monitor the energy storage system’s charging and discharging states. A small-signal model in the converter control system is used [85]. The three-port converter connects a Green power supply, battery backup and charge at the same time. The power flow converter is a fixed energy conversion device that achieves high performance, integration, and power density [86].

The high voltage conversion gain is accomplished in dc/dc converters with multiple inputs and a single output for electric vehicles and their advantages are no limitation for switching duty cycle, less current stress, the control range is more comprehensive for different input powers. If any input fails, it continuously provides energy to the load [87]. For high-power electric vehicle applications, a multidevice interleaved boost is used. It reduces output voltage ripples, input current ripples, volume, and increases the performance when handling high current at the input side [88]. The simplicity of multiport converters can be used to charge batteries in electric cars by using hybrid sources [89]. The topology combines current doubler and buck chopper using galvanic isolation at high-frequency in multiport converter. This converter achieves multiple current path smaller output capacitor and significantly less conduction loss [90]. The validated synchronous digitally operated multiphase multi-switch boost converter is an alternative to interleaved boost converters. In the continuous conduction mode provides part count reduction and size. Its characteristics include low input current ripples, high current capacity, even distribution of power loss, and high performance. This converter is mostly intended for use in the front end and rear end of fuel cell electric vehicle applications [91]. The interleaved multiphase, multi-switch boost converter is mainly intended for use in fuel cell electric vehicle applications. It has enhanced current module-level capabilities by sequential operation of several parallel power and switches. By selecting proper parallel switches, the current sharing problem is avoided [92].

#### 4.1.5 Switched capacitor bi-directional converter

**Fig. 16:**
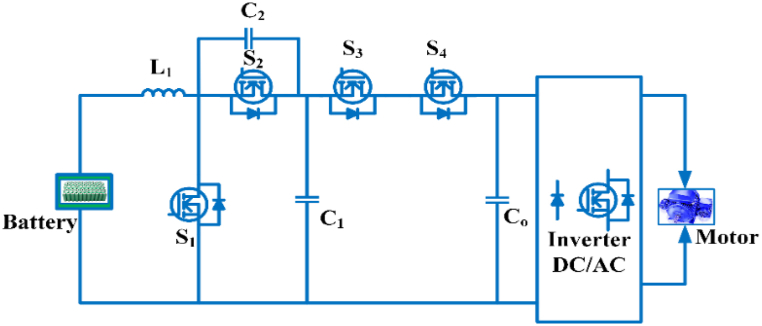
Switched capacitor bidirectional converter.

The switch-capacitor Bi-directional converter configuration consists of four switches, three capacitors, and one inductor, as shown in Fig. 16. As S_1_, S_3_ are switched on, current is passed from the battery storage device to L, and by C_2_, capacitor C_1_ is charged. S_2_, S_4_ are switched ON in step-down mode, while S1, S3 are turned off. C2 charges, followed by C1 being charged from C_o_ [93]. The voltage gain of switched-capacitor Bi-directional converter is expressed as:

(6) $$\frac{V_{o}}{V_{i}}=\frac{2}{1-D}$$

In an electric car, the switched capacitor interleaved bi-directional converter has an enormous voltage gain. The low voltage side features low current ripple, low voltage stress across switching devices, and an input/output common ground [94]. The low voltage side of the bidirectional interleaved switching capacitor dc-dc converter is connected in order to achieve a high step-up/step-down voltage gain. By connecting to a high voltage, it reduces current ripple and achieves a high voltage gain. Besides, without adding extra hardware’s synchronous rectification operation, it is carried out and increases the converter’s efficiency [95]. To reduce the total switch, phase shift switch capacitor integrated with a PWM converter and regulate the load or battery phase shift angle and duty cycle should control [96]. A bidirectional switching capacitor dc-dc converter can be used to control the voltage gain for electric cars equipped with a hybrid energy source. It has several benefits, including reduced part count, increased voltage range, low voltage tension, and common land. Additionally, a synchronous rectifier enables zero voltage switching without the use of additional hardware, thus increasing the performance of the converter [97]. To enhance the voltage, a switched capacitor replaces the pricey inductor with a capacitor. The switched capacitor converter enables higher power density to raise or buck the voltage while still reducing part count and cost. This converter applies to all power conversions [98]. To maintain the voltage balance of battery packs, a combined buck-boost and switched capacitor converter based on equalizers is used [99]. Lithium-ion batteries suffer energy and power loss in cold conditions. A regular operation of an Electric Vehicle involves heating. AC heating systems are unsuitable for use in electric vehicle applications. Specifically, a sine wave heater is used, and its features are low cost, small size and excellent stability and ease of control [100]. Non isolated converters for electric vehicles are compared in Table 9.

**Table 9:** Non-isolated converters for electric vehicles are compared.

Type	DC-DC converter	Objective	Conclusions	Advantages	Drawbacks
Non-isolated converter	CC [[64], [65], [66]]	To avoid a large amount of energy being wasted.	Ensure that the output is smooth and free of ripples.	Inductor’s peak-to-peak ripple current is lower. Continuous input and output current	Stabilization is difficult Resonance is uncontrolled and undampened.
	SCBC [[93], [94], [95]]	To obtain high gain of voltage while also achieving high level of performance	A high level of efficiency above 90%.	economical. Convenient design Current output is limited.	several ripples in the current. Improving efficiency across a broad range of input-to-output voltage ratio is difficult.
	CIBC [69,70]	To minimize ripples in output current and inductor current.	Increased efficiency is achieved by increasing the coupling coefficient.	Size is little. The price is low. Ripples have been reduced.	There is limited possibility of failure. Voltage ripples are not taken into account.
	QZBC [[76], [77], [78]]	Achieve a broad gain range and a perfect common ground.	The maximum and minimum efficiencies are 96.44% and 88.17%	Reduce switch-related stress. Component rating is reduced. Capability to buck/boost.	Input current is discontinuous The capacitor is subjected to a high voltage stress.
	MDBIC [[82], [83], [84]]	To keep the number of passive components to a minimum. To decrease ripples in input current and output voltage. To achieve adequate control and a rapid transient reaction.	Undergoes lower EMI as well as low stress. Decreases current and voltage ripple by half as compared to IBC. Reduces the bulk of the inductor and capacitor by half as comparison to IBC.	Current stress levels are low. Extremely effective. Ideal for conversion of large amounts of energy. A straightforward way to control. Reduced the size of the heat sink and component.	Owing to the large number of elements, the circuit is complex. Under load heterogeneity, the duty period is extremely vulnerable. It’s difficult to study in both steady state and intermittent environments.

### 4.2 Isolated converter (dc-dc) for electric vehicle

#### 4.2.1 Flyback converter

**Fig. 17:**
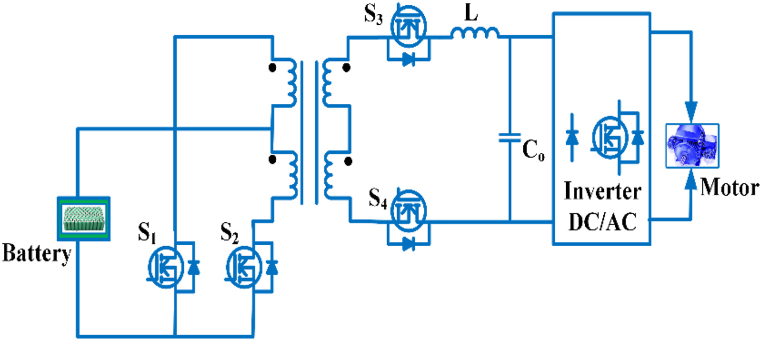
Fly-back converter.

A Flyback converter configures the electric vehicle. It has two switches, two capacitors, and for isolation, the transformer is placed as shown in Fig. 17. When the switch is in ON mode, voltage is created on each side while transformer action takes place [101]. The magnetizing current for the closed switch and open switch operation becomes

(7) $$\Delta i_{Limclosed}=\frac{VsDT}{Lm}$$

(8) $$V_{s}=V_{p}\frac{N_{2}}{N_{1}}$$

(9) $$\Delta i_{Limopen}=\frac{-Vo\;\left(1-D\right)T}{Lm}\frac{N_{1}}{N_{1}}$$

The fly-back converter is often used to transfer between AC and DC as well as DC and AC and is insulated in galvanic form between incoming and output. It’s like a squeeze converter then the inducer is divided into a transformer form. So, a further advantage is the voltage ratio compounded [102]. During on state, energy is stored, and while in an OFF state, it transfers energy [103]. The flyback converter can be operated in variable frequency. Due to its low cost and high separation characteristics, it is mostly used in low power applications. The performance of converter efficiency is affected by high-frequency losses and switching losses [104]. It is used in high load voltage application due to elimination of freewheeling diode and saving the cost by removing the inductive filter in filtered output [105]. Improved numerical model of the flyback converter is designed for Electric vehicle consists of transformer and resistor and integrated circuits [106]. Multipower port topology is used in hybrid electric vehicles to handle multiple power sources and maintain wide load variation, high voltage gain, less ripple current, and modular structure parallel energy battery [107].

#### 4.2.2 Push-pull converter

**Fig. 18:**
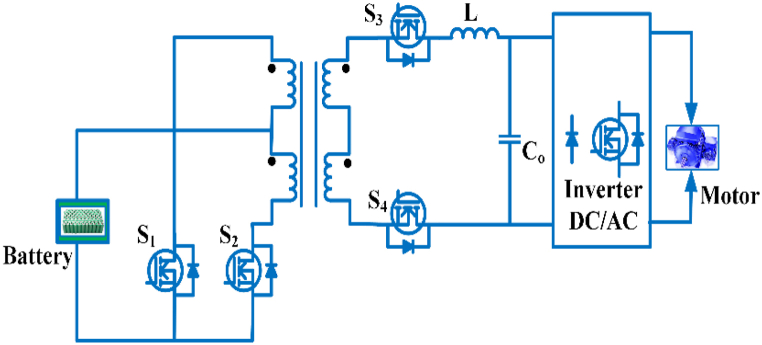
Push-pull converter.

A rectifier diode, condenser and transformer as well as four switches S_1_ S_2_, S_3_& S_4_ at the same time are used for the push-pull conversion for the EV train as illustrated under Fig. 18. The output transformer determines the turn ratio, and the output voltage is regulated by feed-forward by transformer action [108]. The voltage gain is expressed as

(10) $$\frac{V_{o}}{V_{in}}=nD$$

Electric vehicle applications are well-suited to the 3 ∅ push-pull converter. it resolves the converter’s hard-switching issues, and it realizes zero voltage switching. Thus, lowers swapping loss and boosts efficiency [109].

Soft-start mechanism is used to restrict the starting power draw, and a push-pull dc/dc converter with soft start PID configuration is used as power transfer for the auxiliary system in electric vehicles [110].

#### 4.2.3 Isolated zero voltage switching dc converter

**Fig. 19:**
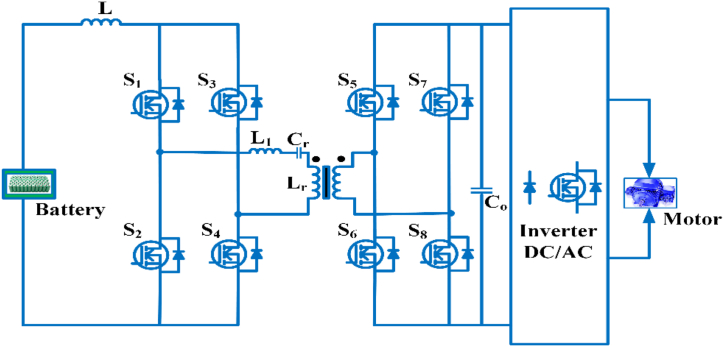
Zero - Voltage switching converter.

As seen in Fig. 19, On the input and output sides of the transformer, the zero-voltage switching converter circuit is a half bridge circuit. The switches and capacitors combined to form a soft switching in the converter. It works in both buck - boost modes [111]. The duty cycle for ZVSC is expressed as

(11) $$\left(1-D\right)\times D=\frac{n\;x\;V_{out}}{2\;V_{in}}$$

A capacitive filter of dc-dc converter is used in the charging of PHEV batteries. It can accommodate soft switching, clamps the voltage through the output rectifier and has a low di/dt ratio, which reduces reverse recovery losses and improves reliability across an inductive output filter [112]. The phase shift zero voltage switching converter is a complete bridge phase shift converter, and more suitable for electric vehicles. The maximum voltage in switches is the same as that of the half-bridge switch as compared to the push-pull converter and not need a center-tapped transformer that it makes it more compact and straightforward circuit [113]. The ZVS Bi-directional dc-dc converter’s topology has the advantage of no total device rating penalty, high reliability, which is utilized in medium-to-high -power applications, such as auxiliary power source in fuel cell vehicles [114]. To reach zero-current switching conditions for the lead lag, an auxiliary transformer is used in a zero-voltage zero-current full bridge converter with insulation transformer. It works from no load to short load, providing high performance [115]. To ensure that the converter operates at zero voltage zero current and to achieve maximum performance. The system relies on a current-driven rectifier to clamp the output diode bridge’s voltage even under all load situations [116]. In the case of hybrid electric vehicles, a bi-directional converter is frequently used to charge and drain a battery stack at the same time. It runs at zero voltage switching and creates a significant voltage diversity, allowing battery bank voltage gain to be transferred to higher voltage level [117].

#### 4.2.4 Resonant converter

**Fig. 20:**
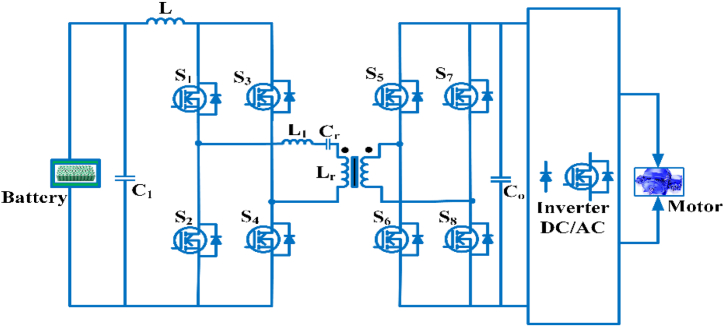
Resonant dc-dc converter.

The architecture of a resonant converter, as shown in Fig. 20. In electric vehicle and vehicle-to-grid applications, the bidirectional resonant dc-dc converter is employed over a huge range of voltage levels, results in great efficiency [118]. The quality factor(Q) and resonant frequency (f_r_) for dc-dc resonant converter is expressed as

(12) $$Q=\frac{\sqrt{\frac{L_{r}}{C_{r}}}}{R_{ac}}$$

(13) $$Fr=\frac{1}{2\pi \sqrt{Lr\;Cr}}$$

The quick charger light electric vehicle includes an AC/DC rectifier and an LLRC with a full bridge, because of its high reliability and minimal switching failure due to zero voltage switching. It is commonly utilized throughout the industry [119].

The unregulated resonant converter topology is in responsible of whole system galvanic separation, using constant current/constant voltage method in buck converter charges the battery within the electric vehicle, and achieving high performance at all times during the battery charging profile [120]. Electric vehicle fast charger benefits from higher operational efficiency from LLC resonant converter. It also reduces conduction by increasing lossless resonance and switching losses [121]. The current and voltage in the circuit are formed using a virtually resonant bidirectional converter. Switching of zero voltage is achieved. In a converter of high frequency, switching loss exceeds power loss soft swapping results in complete failure elimination. As a result, the working temperature and heatsink are lowered. The problem with using GaN switches in bidirectional converters is of high voltage tension, which is handled in an active stacked configuration by connecting the HBGM [122]. The primary switches of a double full bridge LLC resonant converter are used to charge batteries for electric vehicles at constant current and constant voltage. The ZVS and ZCS is achieved in the continuous charge action for current and voltage for all primary switches, which leads to high efficiency [123]. The topology of two-way resonant converter for electric vehicle to grid communication over a wide range of battery voltages, provides a high-efficiency. These switches are hard to turn off both in forward and reverse direction, two bottom switches on the front side are used for Sic- MOSFET. It is a resonant converter for full bridge pulse width modulation and is a resonant half bridge boost converter in the transverse direction. It has benefit of having a broad reverse voltage range [124].

#### 4.2.5 Isolated dc multiport converter

**Fig. 21:**
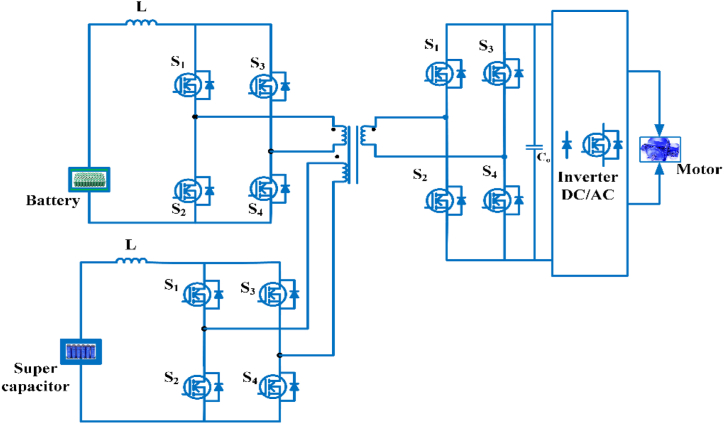
Isolated DC Multiport converter.

The Multiport Isolated Converter is used to restore power from regenerative braking to the input sources. It is combined with multiple input sources, increasing the converter’s performance. The addition of a transformer raises the converter’s weight. As the number of devices increases, synchronization gets more complex as shown in Fig. 21 [125]. The energy flow between the ports is maintained by duty cycle and phase shift regulation, it is used to maximize system operation in order to minimize the overall system failures. The control is primarily applicable to hybrid electric vehicles' multiple voltage electrical systems [126]. An isolated multiport converter can regulate power flow in multiple directions and connect electric vehicles to the grid. A smart microgrid is used to deliver power to loads in order to mitigate the impact of load shedding and increase energy efficiency. This microgrid requires an electric vehicle and other distributed energy storage units [127]. The interleaving technique links the input sources while reducing input current and output voltage ripples [128]. Table 10 compares isolated dc/dc converters; Table 11 compares dc/dc converters generally in electric cars; Table 12 provides specifications for both isolated and non-isolated converters.

**Table 10:** Comparison of Isolated dc/dc converters.

Type	DC-DC converter	Objective	Outcomes	Benefits	Drawbacks
**ISOLATED CONVERTER**	Fly-back converter [[101], [102], [103]]	To accept a wide variety of input voltages	Reduces leakage inductance to an appropriate level.	performance is separated from the primary. Multiple output voltages are possible. Ability to control multiple output voltages.	Ripple current. Losses are higher. More capacitance at the output and input. In the compensation loop is the right half pole.
	Push-pull converter [108,109]	To adjust the DC power supply’s voltage	starting power is limited. On the main line, it achieves low current and voltage.	Transistors and transformers are used more efficiently. EMI is reduced. Filtering isn’t as necessary.	Transformer for the central tap. In the flux walking phenomenon, two switches are rarely used.
	Zero voltage switched capacitor [[111], [112], [113]]	To provide enough power over a wide variety of load variations. To achieve an adequate level of soft-switching reliability. The output diode bridge voltage is clamped.	In all load conditions, achieves zero voltage switching. The symmetric auxiliary circuits ensure a safe and efficient operation under no-load conditions.	EMI is poor. Switching losses is low. There is no need for an additional clamping circuit.	It is necessary to use a large capacitor. Current ratings are High. Fault-tolerance is lacking.
	Resonant converter [[118], [119], [120]]	To minimize magnetic components and passive filters	Achieves a high level of step up/step down capability. Achieves a high level of quality. Offers a broad range of voltage gain	Low cost. Conversion rate is very high. High productivity.	Expensive controller. Complex integrated transformer.
	Multiport Isolated dc-dc converter [125,126]	Optimizing the system’s efficiency by controlling the duty cycle. To reduce overall device losses as much as possible. To look at the complex analysis and the control strategy that goes with it.	Achieves a dynamic response is fast. Power flow can be regulated independently. High efficiency is achieved by duty cycle and phase shift management.	Voltage gain is high. Low ripple current in the output voltage. Isolation by galvanic action.	Under steady-state and intermittent conditions, complex analysis is performed. The duty cycle under load shifts leads to high sensitivity. Synchronization is difficult to obtain.

**Table 11:** Comparison of dc/dc converters in electric vehicles.

DC-DC Converter	Current/Voltage Ripple	Switching Frequency	Complexity of Control Circuit	High power Conversion	EMI Suppression	Cost	Voltage Gain	Active components			Passive components	
								D	SW	HFT	L	C
CC [65,68]	Simple	High	Simple	Appropriate	Reduced	Low	$\frac{-D}{1-D}$	2	2	0	2	2
SCBC [94,98]	Moderate	High	Moderate	Appropriate	Needed	Medium	$\frac{2}{1-D}$	4	4	0	1	3
CIBC [69,72]	Moderate	High	Moderate	Appropriate	Needed	Low	$\frac{2+n-D}{1-D}$	3	3	0	2	3
QZBC [77,80,81]	Simple	High	Complex	Appropriate	Needed	Medium	$\frac{1+D}{1-D}$	3	3	0	2	3
MDIBC [84,[90], [91], [92]]	Complex	Low	Complex	Appropriate	Reduced	Low	$\frac{1}{1-nD}$	16	16	0	4	1
PPC [108,110]	Simple	High	Complex	Appropriate	Reduced	Low	nD	4	4	1	1	1
FC [102,106,107]	Simple	High	Moderate	Not appropriate	Needed	Low	$\frac{nD}{1-D}$	2	2	1	0	2
RC [119,121,124]	Simple	High	Moderate	Appropriate	Reduced	Low	n [j2πfsw]	8	8	1	2	3
ZVSC [113,115,116]	Complex	Low	Complex	Appropriate	Reduced	Medium	$\frac{2}{\pi }$ D (1-D)	4	4	1	1	5
MPIC [126,128]	Complex	Low	Complex	Appropriate	Needed	High	$\frac{n+1}{1-D}$	12	12	1	2	1

CC-CUK converter, SCBC-Switched capacitor bidirectional converter, CIBC-coupled inductor bidirectional converter, QZBC-Quazi z-source bidirectional converter, MDIBC-Multidevice interleaved bidirectional converter, PPC- push-pull converter, FC- Fly-back converter, RC-Resonant converter, ZVSC- Zero voltage switching converter, MPIC- Multiport Interleaved converter

**Table 12:** Overall specification of isolated/non isolated converter.

S.No.	Topology	Fig No.	Vin(V)	Vo (V)	Fs (KHz)	I_Lmax_	ΔI_Lmax_	ΔV_o_	No. of phase	Turns	Po (KW)	D	L (μH)	C (μF)
1	Cuk converter [67,68]	12	160 260	300	20	–	–	–	–	–	0.85	0.29 0.39	299.67	1.32 2000
2	Bidirectional converter with coupled inductor [71,72].	13	48	380	40	–	–	–	–	1:1	3.2	0.5	200 800	27 270
3	Bidirectional converter with Quazi Z source [[79], [80], [81]]	14	30	200	100	6.5	–	–	–	1:1	0.4	0.57	300	10 100
4	Multidevice interleaved bidirectional converter [[86], [87], [88]].	15	250, 200	400	20	100	10	4	3	–	30	0.5	187 160	160
5	Switched Capacitor bidirectional converter [[96], [97], [98]]	16	48	400	400	10	–	2.5	–	–	30	0.5	100	1000
6	Fly back converter [106,107]	17	200	400	40	75	3.75	4	1	1:2	30	0.5	1200	14.64
7	Pull converter [110]	18	200	400	10	15	–	–	–	3:5	1	0.5	780 300	2500
8	Isolated zero voltage switching converter [[114], [115], [116]]	19	100	300	20	–	–	3	1	1:3	1.6	0.35	560	10
9	Resonant Converter [[120], [121], [122]]	20	150	380	30	7.5	0.75	4	4	–	5	0.5	6670	25
10	Isolated multiport converter [127,128]	21	280	400	20	–	–	–	–	1:2	30	–	175	150

## 5 Fast charging techniques

Charging and discharging affect battery performance, safety, and durability. Each charging technique for LIBs in EVs has benefits and drawbacks. These charging techniques vary in charging time, efficiency, battery temperature, lifetime, SOH, energy loss, and deterioration. Below are some current quick charging techniques. Every charging technique affects battery life [129].

### 5.1 Constant current charging method (CC)

**Fig. 22:**
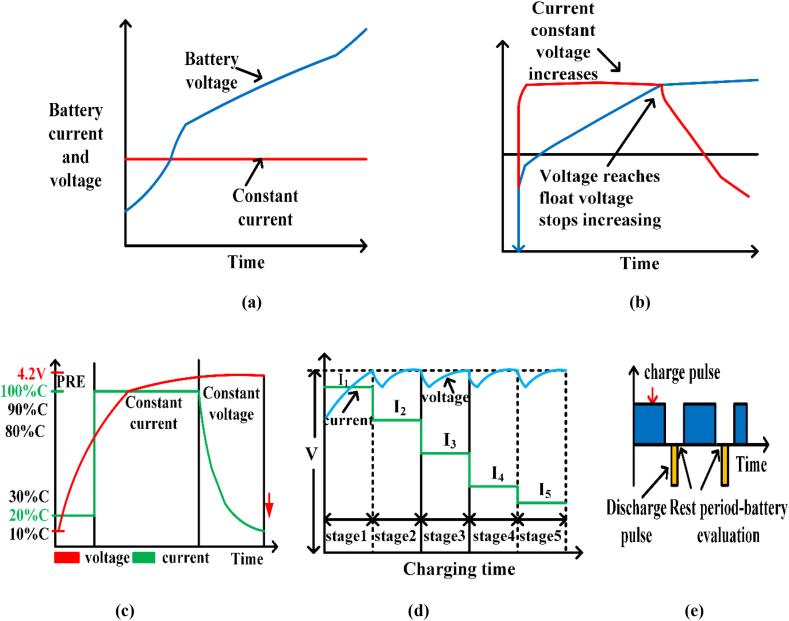
(a). Constant current charging; (b). Constant voltage charging; (c). Constant current and constant voltage charging; (d). Multistage Constant Current Charging; (e). Pulse charging method.

The primary and frequently utilized CC battery charger is rugged. Fig. 22a shows this way of charging the battery at a tiny constant C-rate. When V_b_ reaches its pre-set value, this procedure stops charging. First used to charge NiCad and NiMH batteries, and subsequently LIBs.

This method is charging the current rate determines battery function. The hardest part of this strategy is choosing a charge C-rate that balances capacity usage and battery charging speed. Using high current rates (C-rates), CC charging speeds up but accelerates battery aging. Low charging current rates allow high-capacity use but reduce battery performance and decrease EV use. Overcharging permanently ruins the battery; hence this CC charging method demands a 100% fully charged detector. This is not easy to implement and increases system complexity and expense. This charging method causes undercharging, overcharging, energy loss, and excessive temperatures. Overcharging damages both electrodes and increases energy loss and temperature [130].

### 5.2 Constant voltage charging method (CV)

This method avoids overcharging by charging the CV to adjust the battery voltage. As shown in Fig. 22b, this charging technique reduces the battery charging current until it reaches the specified threshold. Early charging requires a high current flow to maintain the controlled terminal voltage. However, this fast charging rate increases the battery’s temperature, resulting in grid breakdown and increased heat loss. The key challenge with this charging technique is finding the optimal constant voltage value to balance charging speed, battery capacity use, and electrolyte breakdown. CV charging speeds up charging but damages batteries and wastes energy. It causes a high charging current and damages the SOC while charging the battery from low voltage. This CV approach’s initial charging current rate exceeds the battery’s acceptance value, collapsing the battery lattice and pulverizing the active ingredient in the battery electrodes. CV charging is rapid because it maintains the average battery current. CV charging is slow, which limits battery charge. CV charging rapidly affects battery life owing to electrolyte oxidation and high initial charging currents [131].

### 5.3 Constant current and constant voltage charging method (CCCV)

In Fig. 22c, this charging method lowers the battery charging current until it hits the threshold. Early charging requires a solid current to sustain terminal voltage. However, quick charging raises battery temperature, causing power outages and heat losses. Two charging stages—constant current (CC) and constant voltage (CV)—are used (CV). If the battery SOC is below the specified threshold value (say 1.2 V), the charger operates in trickle current (TC) mode with a low charging rate (0.1 C) to minimize deep discharge damage. Fig. 22c shows the charger switching from TC to CC mode when the battery SOC exceeds 1.2 V. TC pre-charging mode. LIB chemistry determines the CC stage’s current rate and threshold [132]. CC mode charges the battery at a constant current until it hits the higher cut-off voltage, such as 4.2 V. When VB Equals 4.2 V, the charger automatically switches from CC to CV. CV stage extends battery charging time by gradually reducing the charging current until it reaches the cut-off current [133]. The CV stage maintains terminal voltage and lowers cell overvoltage stress. CCCV charging takes longer but charges almost wholly. The CC phase current rate determines the charging duration, which ranges from 1 to 2.5 h. A low charging current rate in CC mode gives us a longer battery life, excellent charging efficiency, and a longer charging time [134]. This CCCV approach may employ a high current rate to shorten the charging time, but the battery’s internal IR voltage drop increases the CV phase Due to the cell’s IR drop, the charger shifts to the CV stage before the cell reaches 4.2 V, increasing the charging time to charge the battery completely. To reduce charging time, compensate for internal IR drop voltage or move the reference value slightly beyond the specified threshold value so cell voltage hits 4.2 V and the charger switches to CV mode. Thus, CV mode charging time improves, reducing CCCV method charging time [135].

### 5.4 Multistage constant current charging method (MSCC)

Internal IR voltage loss in the CC step of the CCCV approach makes charging LIBs difficult. The conversion period from the CC to the CV stage affects charging time and battery degradation [136]. CCCV can cause overcharging and extended charging times. MSCC addresses these CCCV issues. MSCC charges LIBs quickly and easily. The CV stage follows two or more CC phases [137]. Fig. 22d shows that each CC stage’s charging current rate decreases (IC1 > IC2 > IC3), showing the five MSCC phases. Each step charges the battery at a lower constant current rate. The charger enters the next CC step when the battery voltage exceeds a threshold. Algorithms determine this threshold value. MSCC employs Particle Swarm Optimization (PSO), Taguchi, Fuzzy Logic Controller (FLC), Ant Colony System (ACS), Genetic Algorithm (GA), Taguchi Orthogonal Arrays (TOA), and others to optimize charging current rate. CC rate charging will decrease till it hits 4.2 V under minimal current. This approach charges faster than CCCV at the same beginning charging current rate [138].

### 5.5 Pulse charging (PC) method

PC charging reduces the main problem of conventional charging: lengthy charging time. Fig. 22e shows this PC approach charging the battery with pulses and rest intervals. In this charging approach, rest intervals remove concentration polarisation at the electrode/electrolyte interface, distribute ions evenly, and reduce lithium plating. This charging technology reduces charging time, enhances energy and charge efficiency, extends battery longevity, and degrades materials with low heat [139]. The rest intervals between pulses evenly distribute ions in the electrolyte, so the next charge pulse absorbs the ions, increasing power transmission and reducing charging time Advanced battery-charge systems utilize this charging approach. Pulse charging settings must be carefully selected to maximize battery longevity and performance. Pulse charging eliminates the risk of charging batteries at low temperatures. Many researchers have created pulse charging techniques (PCMs) that employ high peak charging voltages and currents, reducing the charging schedule compared to the CCCV approach. Controlling pulse duty cycle, frequency, and amplitude improves charging [140]. Table 13 compares battery charging in electric vehicles, while Table 14 compares charging methods qualitatively.

**Table 13:** Comparison of battery charging approaches in EVs.

Approach	Advantages	Disadvantages	Key elements
CC [130]	Easy to implement	Capacity utilization is low	Charging constant current rate Terminal condition
CV [131]	Easy to implement Stable terminal voltage	Easy to cause the lattice Collapse of battery	Charging constant voltage Terminal condition
CC-CV [132,133]	Capacity utilization is high Stable terminal voltage	Difficult to balance objectives such as charging speed, energy loss, temperature variation	Constant current rate in CC phase Constant voltage in CV phase Terminal condition
MCC [[136], [137], [138]]	Easy to implement Easy to achieve fast charging	Difficult to balance objectives such as charging speed, capacity utilization and battery lifetime	The number of CC stages Constant current rates for each stage

**Table 14:** Qualitative comparison of charging methods [130,131,134,135,139,140].

Methods	CC	CV	CC-CV	PC
**Life cycle**	Low	Low	Medium	Low
**Control Complexity**	Low	Low	Medium	Medium/high
**Charging Efficiency**	Low	Low	Medium	Medium
**Charging time**	Medium	High	Medium	Medium

## 6 Charging station configurations

Sustainable plug in electrical vehicle (PEV) charging stations use multi-energy Renewable energy system (RES) to generate power. Hybrid charging stations operate outside the grid. These systems need storage technology to fulfil load demand at night or during peak periods. These separated charging stations offer auxiliary services and power fluctuation protection [141]. Researchers designed remote charging stations with various energy storage system (ESS) designs [142]. The ESS in isolated hybrid PEV charging stations typically has three variants.

### 6.1 Single energy storage system configuration

**Fig. 23:**
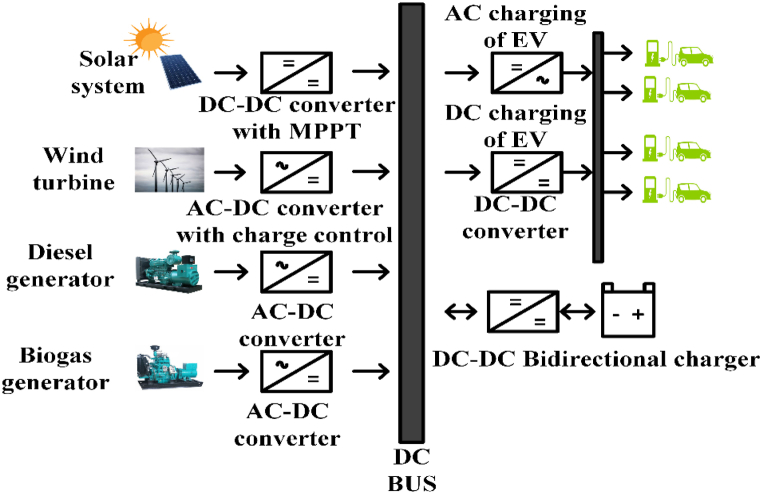
Standalone single energy storage system.

This setup supplies power utilizing locally accessible renewable energy resources and a single energy storage device. Due to having only one sort of storage, these systems are simple and cheaper. Fig. 23 shows the basic construction of this design. This system may employ a diesel generator to generate electricity from renewable sources in an emergency. Li-ion batteries are more efficient and cost-effective in a single-storage design [143]. The battery evolves from lead acid to nickel-based and lithium-ion [144]. The battery charging time and energy cost depend on an electric vehicle’s arrival time. Considering EV arrival time and state of charge, the authors constructed a wind, solar, and Li-ion battery-based plugin electric charging station. Using a genetic algorithm, the design was optimized. Compared to a traditional grid design, a standalone hybrid system with a single storage configuration requires less investment and generates more profit from delivering power to electric automobiles. Standalone systems have higher battery replacement costs than others but do not pay for grid electricity [145,146].

### 6.2 Hybrid energy storage system configuration

**Fig. 24:**
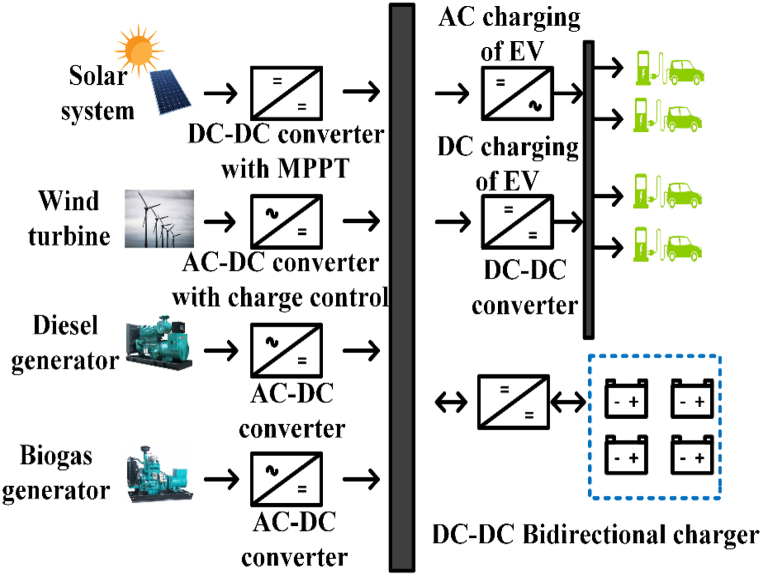
Hybrid energy storage system.

The critical drawback of renewable energy (RE)-based hybrid systems is the energy storage devices' short lifespan [147]. Researchers suggest hybrid energy storage systems. This combination improves storage capacity and economics depending on RE resources utilized for power generation. High-power storage systems deliver high power for a short time, whereas high-energy storage devices supply average power over a longer time. High power and energy storage technologies yield the most significant economic returns [[148], [149], [150]]. The plugin EV may store surplus electricity during off-peak hours and return it to the charging station during peak hours, helping other local energy storage systems. Fig. 24 shows the fundamental system structure. The standalone microgrids use battery and supercapacitor storage. A hybrid system is used because batteries store energy, and supercapacitors store power and also suggested another two-layered hybrid energy storage system. This system combined Li-ion and lead acid batteries for typical loads and supercapacitor-based storage for abrupt power fluctuations [151]. This paper presented a freestanding hybrid EV charging station using Li-ion batteries, hydrogen, and ammonia-based storage to charge 50 vehicles per day [152].

### 6.3 Swappable storage system configuration

**Fig. 25:**
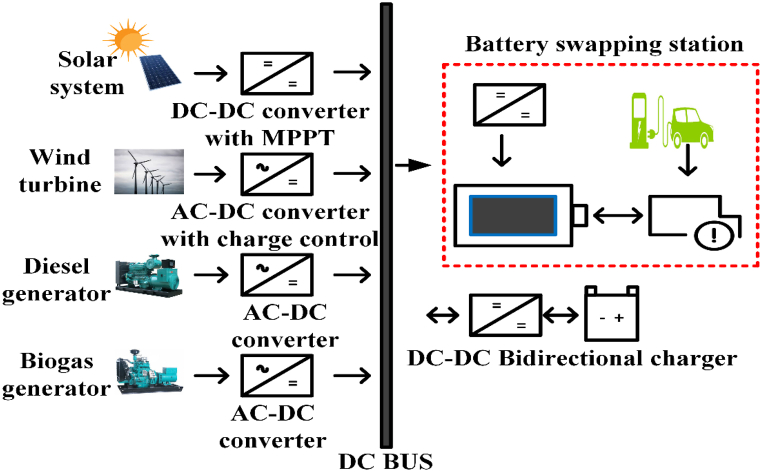
Swappable energy storage system.

Electric vehicle (EV) charging system technology includes swappable storage. The charging station’s fully charged battery replaces the EV’s low battery. In the battery-swapping charging station architecture, EVs enter and depart a charging station area. It has a battery stacking unit to receive the battery and a battery replacement robot mounted in the charging station area to replace the battery. A data acknowledgment unit collects data about the EV that enters the charging station, such as type, size, charging state, delivery date, charging date, and battery type. Its charging station control unit controls the battery-switching robot. Fig. 25 shows a simple charging station structure. The biggest obstacle to EV adoption is the time it takes to charge an EV at a specialized charging station. Battery charging stations are another option. Swapping stations must standardize EV batteries and build a commercial strategy for pricing allocation and operation and developed a rigorous economic comparison approach to assess battery-swapping station setup costs with optimized battery-swapping station planning for EV owner satisfaction [153,154]. Zubaida Fakhruddin Khan and Rajesh Gupta created a wind-powered EV battery-swapping station. These batteries enable wind backup and vehicle storage. Van Ga Bui et al. suggested hydrogen cylinders for two-wheeler EV storage [155]. Steffen Schmidt suggested a battery swap loan program to increase economic and environmental benefits [156]. This vehicle has a battery charge management location and communication system. It can transport batteries from a RE-powered remote charging station to EVs [157]. EV owners are not compelled to buy Li-ion batteries. EV owners lease these batteries [158]. A. Rezaee Jordehi et al. placed freestanding battery-swapping stations with PV, wind, and geothermal power and plug in hybrid energy storage (PHES) [159]. A bi-directional approach by Yang Li et al. reduced the cost of an isolated RE-based microgrid and increased the profit of its battery-swapping station Energy management can reduce the cost of battery replacement and charge in a freestanding battery-swapping station [160]. Table 15 shows battery configuration in recent research work, and Table 16 shows an overview of EV charging configuration.

**Table 15:** Battery configurations in recent research work.

Battery configuration	Resources	Storage type	Merits	Demerits
Single storage [[144], [145], [146]]	Wind and solar	Li-Ion battery	High energy density	Li-ion battery capital costs are very high.
	Solar and Biogas	Lead acid battery	Decreases in CO2 emissions of 34.68% when solar energy is combined with biogas and lead-acid batteries for storing	Adverse effects of lead-acid batteries on the ecosystem.
	Wind and solar	Fuel cell	Extended storage life.	hefty storing costs for hydrogen.
Hybrid Storage [[147], [148], [149], [150], [151], [152]]	Wind and solar	Li-ion battery, Hydrogen, Ammonia storage	Economical operation of the outlying station for 50 car charging.	Li-ion batteries have a restricted amount of charging cycles. Store with a limited energy density.
	solar	Li-ion battery and super capacitor	SC is used to extend battery life. Design of the economic structure. • Durable energy storing • Lithium-ion batteries have a higher energy storing capacity than lead-acid batteries.	• The capacity of storage increases, and SC is only used to manage power variations.
Swappable storage [[153], [154], [155], [156], [157], [158], [159]]	Solar	Li-ion batteries	improved generation and ESS sizes. The issue with the prolonged recharge times is resolved.	There is no particular energy control strategy. Only Photovoltaic production increases in costs.
	Wind and solar	Li-ion batteries	Bi-level scheduling optimization between the isolated microgrid and battery swap station. Maximising profits.	There is no consideration of the microgrid’s reactive power.

**Table 16:** Overview of EV charging configurations [145,149,151,156,158].

EV configurations	Merits	Demerits
Only grid power is used at the charging station.	Operating expenses will be less. It can assist with peak load levelling.	Oversaturation of the grid. Infrastructure for charging is required at a large capital cost.
Charging station that uses grid power and a system for storing energy	Adaptable operation in the face of failure. The owners will benefit from ESS.	Systemically difficult Grid integration is a significant disadvantage. Capital expenditures are substantial.
Charging station using grid power, renewable energy, and energy storage	The operating cost is too inexpensive. Convenient for charging multiple sources simultaneously.	Intricate in operation. Developing a system with efficient control algorithms will necessitate a greater financial investment. Identifying a defective system is challenging.
Technologies for battery swapping	The battery cells' durability will increase.	Initial establishment costs are substantial. Infrastructure for charging is required. The voltage, energy density, and shape of batteries need to be standardized by global OEMs.

## 7 Recent trends and challenges in EV fast charging

**Fig. 26:**
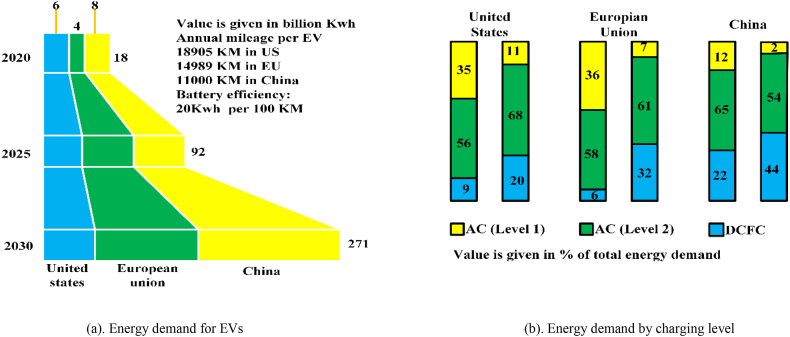
(a). Energy demand for EVs; (b). Energy demand by charging level.

EVs will drastically increase energy consumption. Fig. 26a shows the three key markets' EV energy demand trend [44,161]. As shown in Fig. 26b, DCFC adoption will expand dramatically but slow ac charging will remain dominant through 2030.

FC guidelines are rapidly developed to aid UFC. CCS (EU), CHAdeMO (Japan), and GB/T (China) are the most common FC standards. The three FC standards are compatible with worldwide AC and DC charging modes (IEC 62 916, IEEE 2030.1.1, and SAE J1772). Tesla’s FC standard is exclusive to the Tesla Supercharger and EVs. Table 17 shows that GB/T 20 234.3–2015 has the lowest maximum charging power at 185 kW and CHAdeMO the highest at 400 kW. To fulfil the requirement for increased charging power, CHAdeMO and China Electricity Council launched ChaoJi, a new standard compatible with GB/T and CHAdeMO. CCS and IEC are helping the ChaoJi workgroup comply [162,163].

**Table 17:** Status of Fast charging standards [162,163].

Standard	CHAdeMO	GB/T	CCS 1	CCS 2	Tesla	Chaoji
**Connector**						
**Inlet**						
**Maximum voltage (V)/current (A)/power (KW)**	1000/400/400	750/250/185	600/400/200	900/400/350	500/631/250	1500/600/900
**Maximum market power (KW)**	150	125	150	350	250	N. A
**Communication protocol**	CAN	CAN	PLC	PLC	CAN	CAN
**Start @**	2009	2013	2014	2013	2012	2020

DC fast chargers (DCFCs) charge at 50 kW moving towards ultra-rapid charging. Mainstream EV batteries average 60.1 kWh (317 km range). Drivers prefer 15 min over overnight charging. Table 18 displays EV models' 15-min charging electricity. The long-range EV charges at 228 kW. Battery capacity increases charging power. 350 kW DCFC meets demand [[164], [165], [166]].

**Table 18:** Market statistics for electric vehicles in 2022 [164,165].

Model	Battery capacity (KWh)	Range (KM)	Average Charging Power (KW)	Actual Maximum Charging Power (KW)
**Nissan Leaf**	39	235	40	46
**Renault Kango E-Tech Electric**	44	215	50	80
**Audi Q4 e-tron 35**	52	285	40	118
**Hundai Kona Electric**	65	400	70	100
**BMW ix3**	74	385	104	155
**Volkswagen ID.4 GTX**	77	400	115	175
**Ford Mustang Mach- E GT**	91	425	86	107
**Tesla Model S Plaid**	95	550	140	250
**Volvo EX90 Twin Motor**	107	455	150	250

Note* - Required Averaging charge power to recharge the EV’s battery from 10% SOC to 80% SOC in 15 min.

### 7.1 Architecture of extreme fast charging stations

**Fig. 27:**
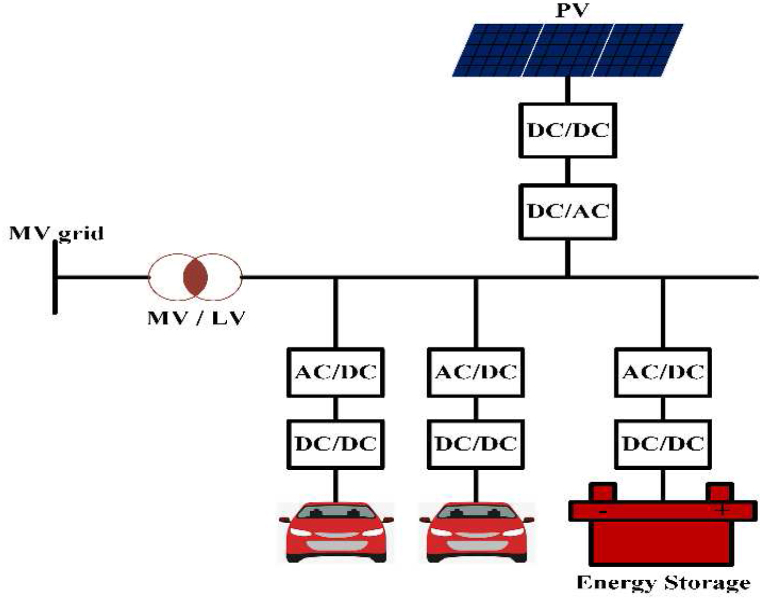
AC connected system.

Fig. 27, Fig. 29 show that the local distribution network between several chargers, local Renewable energy system (RES), and energy storage can be either ac or dc. The advantages and difficulties of each strategy are listed in Table 19. The following sections discuss these issues, possibilities, and the two types of charging stations' implementation strategies [167].

**Table 19:** Comparison of AC and DC connected system [168].

	AC	DC
Conversion stages	More	less
Front end de-rating	Lower	Higher
Control	Complex	Simple
Metering	Standardized	Complex
Protection	Straightforward	Complex
Efficiency	Lower	Higher

#### 7.1.1 XFC stations with AC connected system

For systems that use AC power, a step-down transformer links the distribution network to a three-phase AC bus with a line-to-line voltage of 250 V–480 V. At the station, each converter has its own AC/DC stage and gets power from the AC bus. This method dramatically increases the transfer steps between the distribution network and the EV or RES dc port. When more conversion steps are added to an AC-connected system, it works less well, making the system more complicated and expensive. The availability and maturity of rectifier and inverter technology, ac switchgear and protective devices, and well-established standards and practices for ac power distribution systems are all benefits of utilizing the ac bus. Most Extreme fast charging (XFC) stations are ac connected systems.

**Fig. 28:**
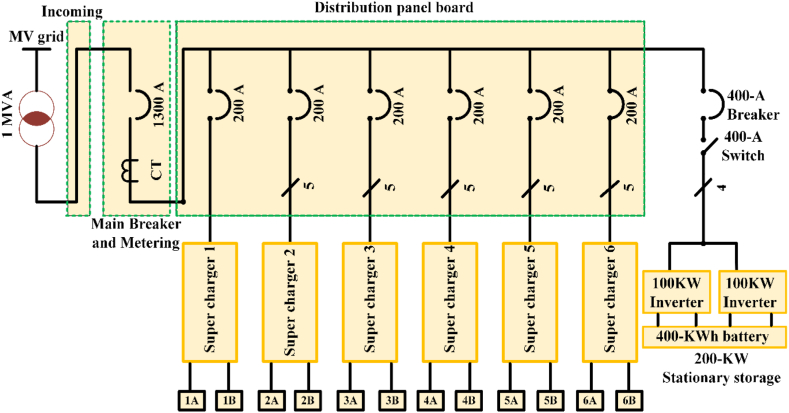
Single line diagram of Tesla super charger station.

Fig. 28 shows the Tesla supercharger station in Mountain View, California. The ABB dc fast charging station in Euroa, Victoria, Australia [169].

**Fig. 29:**
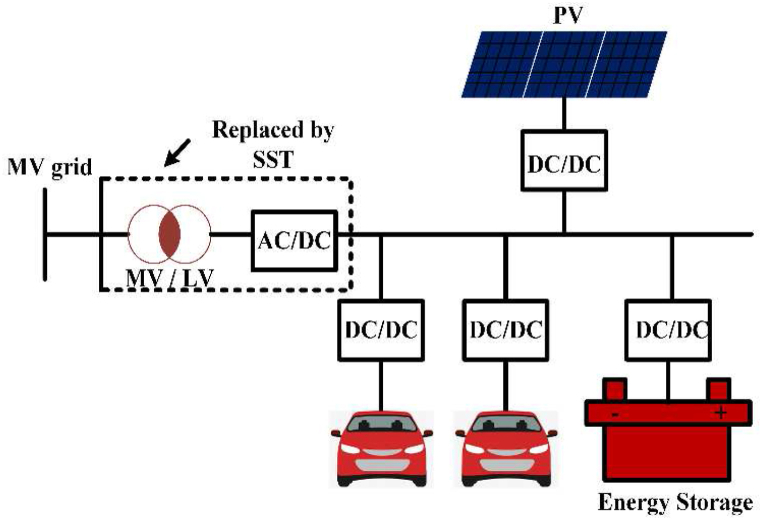
DC connected system.

#### 7.1.2 XFC stations with DC connected system

One central front-end AC/DC converter is utilized to generate a dc bus for dc-connected system, offering a more energy-efficient method of connecting dc energy storage and renewable energy sources. A low-frequency transformer is found at the middle front end, followed by an SST or an LV (250 V–480 V) rectifier stage that performs isolation, voltage step-down, and rectification in a single unit. The dc bus voltage is typically less than 1000 V to fit the current battery voltage range, which is about 400 V. The design of XFC stations with a dc bus should adhere to the same criteria as XFC stations with an ac bus at this voltage level. Instead of using separate AC/DC converters, each charger is interfaced with the dc bus using a DC/DC converter [170].

Typically, the dc bus voltage is less than 1000 V to meet the 400 V battery voltage range. XFC stations with a dc bus at this voltage level should adhere to the same design criteria as XFC stations with an ac bus. In replacement of different AC/DC converters, each charger is connected to the dc bus via a DC/DC converter. Compared to ac-connected systems, systems with fewer conversion stages are more efficient. The “dc distribution” strategy may benefit from a central front end with a singular connection to the utility. Utilize the load diversity created by varying EV battery capacities and charge acceptance as a function of state of charge (SOC) to considerably reduce the cost of the AC/DC converter and the grid connection nameplate. It will reduce the system’s overall installation costs. The absence of reactive power in DC systems, which facilitates control, is an additional benefit of the system. The connectivity of the single inverter to the main grid facilitates isolating from and connecting to the grid. Using partial power converters as the interface between the DC bus and the vehicle may be an additional advantage of dc distribution systems [168] The grounding configuration, fault type, system topology, component specification, size, etc., all play a complicated role in the coordination of protection for dc-connected systems. If the charges are bidirectional, this issue becomes considerably complex. A dc-connected system is susceptible to disturbances since it has low inertia and might become unstable if a fault is not quickly cleared. Consequently, a system’s ability to be restored depends on how quickly faults can be found and isolated. Studies on current dc power distribution systems, including LV dc microgrids, guide the protective coordination of dc-connected charging stations. Considering the cooperation between various protective devices, a protection approach for an LV dc microgrid is provided. Dc systems with a loop-type bus are addressed. The suggested system can locate and isolate the fault and provide power uninterruptedly [171]. In order to monitor energy generation and utilization of RES, battery energy storage, and EV stations in a dc-connected system, dc meters must be installed. This data is necessary for users of EV charging stations to get appropriate invoicing, and it may also be used to plan future station locations. Although dc meters are sold commercially, no recognized accuracy, calibration, or testing standards permit these devices to be utilized for metering. Developing such standard and certified dc meters for dc-connected systems is essential [172].

## 8 Charging technology for heavy duty electric vehicle in European Union

Currently, a diverse array of chemistries and designs are readily accessible and appropriate for electric vehicles (EVs).

### 8.1 Legal framework for charging infrastructure in the EU

Directive 2014/94/EU, also known as the Alternative Fuels Infrastructure Directive (AFID), introduced the incorporation of technical requirements and legal regulations on charging infrastructure for electric vehicles. As per the AFID (Automotive Fuel Industry Association), a projection was made by 2020. There would exist a ratio of one publicly accessible charging point for every ten registered electric vehicles. The AFID (Alternative Fuels Infrastructure Directive) was officially repealed in 2021 and replaced by a new regulatory framework known as the Alternative Fuels Infrastructure Regulation (AFIR). The objectives of the Alternative Fuel Infrastructure Regulation (AFIR) are legally binding for European Union (EU) Member States. The initiative aims to ensure the establishment of a fair and equitable alternative fuel infrastructure across the European Union. The AFIR (Automotive Future Impact Report) suggests that as the electric car fleet grows, there will also be an increase in the capacity of public charging infrastructure. The Alternative Fuel Infrastructure Regulation (AFIR) sets forth specific and stringent requirements for alternative fuel infrastructure.

The policy establishes a specific timeframe for achieving infrastructure objectives, outlines the minimum distances that must be maintained between charging points, and outlines the necessary power and capacity requirements for charging points. According to the AFIR assumptions, it is advisable to position charging stations for light vehicles on the TEN-T network at a minimum distance of 60 km from each other. By 2025, it is anticipated that each station will have a combined capacity of 300 kW. It is also necessary to ensure that every station is equipped with at least one charging point with a minimum capacity of 150 kW. According to projections, the total power output of the stations is expected to double by the end of 2030, reaching a capacity of 600 kW.

Furthermore, there will be a doubling in the number of points that can deliver a minimum power output of 150 kW. The charging stations for heavy vehicles will be strategically located at regular intervals of 60 km. By the year 2025, it is anticipated that all stations will have a combined capacity of 1400 kW [173,174].

Additionally, each station must incorporate a minimum capacity of 350 kW in at least one charging point. The anticipated power capacity goal for the station by the end of 2030 is established at 3500 kW. Furthermore, ensuring that the station includes at least two points with a power capacity of 350 kW is necessary. The requirements for the comprehensive Trans-European Transport Network (TEN-T) have been synchronized with those of the base network, although with extended timelines for implementation. The implementation of the comprehensive network is anticipated to reach full completion by the conclusion of 2030, whereas the base network is forecasted to be finalized by 2035. The AFIR system outlines the specifications for hydrogen refueling stations on the TEN-T core network. As per the specified objective, it is anticipated that by the year 2030, hydrogen refueling stations will be conveniently available at regular intervals not exceeding 200 km. As specified in the reference, the stations will have dispensers with a minimum pressure capacity of 700 bar. Under the stipulations detailed in Fit for 55, Member States must submit a project to the European Commission by 1 January 2024. This project aims to provide a comprehensive overview of the necessary infrastructure and framework required to facilitate the development of the domestic alternative fuels market in the transportation sector. Directive (EU) 2018/844 provides a comprehensive overview of the technical guidelines and legal regulations regarding charging stations for electric vehicles in the European Union. By the directive mentioned above, the member states of the European Union (EU) must meet the following obligations: Enable the creation of easily accessible infrastructure that will efficiently reduce the costs associated with installing charging points for individual vehicle owners. They are granting electric vehicle users the requisite access to charging points. When integrating electromobility requirements into national legislation, it is crucial to consider several factors. These factors include the ownership of buildings and adjacent car parks, the participation of private entities in the management of public car parks, and the varied functions of different facilities, both residential and non-residential [175].

### 8.2 Battery development

Currently, a diverse array of chemistries and designs are readily accessible and well-suited for electric vehicles (EVs). Continuous progress is being achieved in developing diverse chemistries for Li-ion batteries for buses and trucks. The specific application of the vehicle should determine the selection of battery technology, as it directly influences the decision-making process for the charging solution. Use cases that demand fast charging necessitate the utilization of batteries and management systems specifically engineered for this purpose. Nickel-manganese-cobalt (NMC) batteries are widely recognized as highly suitable for opportunity charging applications. There is ongoing development and testing of NMC modular battery technology for articulated electric buses. This battery technology has a capacity of 640 kWh. The batteries are expected to commence series production in the first half of 2021. Lithium iron phosphate (LFP) is a viable alternative technology currently available. It has been specifically engineered to cater to the needs of larger-capacity battery packs. It is essential to highlight that Lithium Iron Phosphate (LFP) batteries provide enhanced safety features compared to Nickel Manganese Cobalt (NMC) batteries. The development efforts are aimed at increasing the capacity and improving the life cycle of batteries and incorporating scalable, modular, and lightweight designs.

In recent years, there has been a substantial reduction in the yearly expenses associated with traction batteries, coupled with noteworthy improvements in their overall performance. The abovementioned factors have significantly contributed to the heightened market acceptance of these products and commodities. There is currently ongoing development of multiple battery chemistries for the next generation. The options encompass semi- or fully-solid-state batteries that use advanced composite or Li-metal anodes and other ionic systems. The technologies mentioned below currently have a lower Technology Readiness Level (TRL). However, they promise to improve the competitiveness of battery-powered electric trucks and buses in the medium to long term. Original equipment manufacturers (OEMs) articulated buses with a maximum battery capacity of 400 kWh. The buses have been specifically engineered to accommodate depot and fast charging capabilities during their routes. Based on the operational characteristics of low-speed travel over short distances, the existing battery capacity is anticipated to adequately fulfil the articulated buses' requirements in the foreseeable future [176].

### 8.3 Charging power

The terms “fast-charging” and “high-power charging” can be ambiguous when referring to HD-EVs, which have different traction battery capacities and voltages than LD-EVs.

Table 20 presents the proposed classification for HDV charging and offers a comprehensive overview of the terminology employed to describe charging power and capacity values. The battery C-rate is a metric used to quantify the rate at which a battery charge. It is closely tied to the battery capacity C, typically denoted in Amperes (A) or, more commonly, in Ampere-hours (Ah). The C-rate is commonly denoted as 1/h and serves as a measure to indicate the rate at which a battery is charged. The C-rate can also be defined as the ratio between the charging power and the battery capacity, commonly called the CP-rate. Various enabling factors support the development of fast charging. The study investigated a range of infrastructural and economic factors that require increased consideration when implementing charging stations with a capacity of 400 kW and above. The critical factors of utmost significance encompassed standardization, coordination, security, grid resources, power demand peaks, and costs [173,177].

**Table 20:** Terminology associated with Heavy duty vehicle conductive charging and the corresponding C-rates [173].

Term	Slow HD-EV charging	Normal HD-EV charging	Fast HD-EV charging	Ultrafast HD-EV charging
**Charging Voltage**	400 VDC, 800 VDC	400 VDC, 800 VDC	Up to 1.5 KVDC	Up to 1.5 KVDC
**Charging current**	60 A–400 A	200 A–800 A	300 A–1 KA	800 A–3 KA
**Charging power**	50 KW–150 KW	150 KW–400 KW	200 KW–1 MW	1 MW–4.5 MW
**Battery capacity**	50 KWh–250 KWh	50 KWh–250 KWh	100 KWh–500 KWh	250 KWh–1 MWh
**C-rate of Charging**	0.2C–1C	0.5C–2C	0.5C–2C	4C–10C

## 9 Conclusion

This review focuses on power electronic converters for electric vehicle drivetrains and provides detailed information and study. This article discusses the architecture aspects, operation core characteristics, advantages, and drawbacks of different advanced and topologies of possible dc-dc converters for use in electric vehicle drivetrains' converters provide a wide range of voltage gains in non-isolated converters, but their cascaded structures limit their power conversion efficiency. While the switched capacitor bidirectional converter enhances conversion performance, it is limited by its high ripple current. Although the coupled inductor bidirectional converter is compact and has a low output current, it suffers from leakage inductance. Although the quazi z source bidirectional converter often has high gain in voltage, it puts a lot of strain on the capacitance. Thanks to its ability to achieve low ripple current and voltage, high reliability, durability, and high-power handling capability, the multidevice interleaved bidirectional converter has become the favored choice for electric vehicle applications.

In case of Isolated converters, the fly back converter has ability to control multiple output voltages; it also has a higher EMI and ripple current rating. While the resonant converter offers superior operation and performance, it requires a complex transformer design and has a restricted capacity for magnetizing current. Despite its low switching loss and EMI, the zero-voltage switching capacitor has drawbacks in terms of high current ratings and fault resistance. Despite the significant voltage gain of the multiport interleaved converter, it comes with drawbacks of a higher part count, ripple current, dynamic inspection, and high sensitivity.

This article outlines the converter configuration for rapid charging in electric vehicle application. To eliminate harmonics in the output current, the Vienna rectifier has a superior responsiveness due to its high power-factor in case of ac-dc converter. Multiple interleaved buck converter provides suitable option for high efficiency with simple design and control techniques in the event of dc-dc converter. Despite the fact that due to its high overall harmonic distortion, this design is inefficient. Based on the articles that were extracted, we have identified that there is a growing tendency toward the utilization of a variety of ways to optimize energy storage system. At this time, technical advancement is moving at a rapid pace in the direction of hybrid energy storage. The research gap identified in the existing research that can be filled by additional investigation in the future.

This article critically examined commercially available EV charging methods. Each charge technique uniquely tackles optimum charging, yet each has perks and downsides. The pulse charging strategy seems best for quick LIB charging concerns. Most significantly, this work provides crucial insights for future research to build designed fast charging processes related to the pulse charging method to address the fundamental disadvantages of current charging procedures. For safe, efficient, and quick charging, battery temperature control should be prioritized.

This review demonstrates remote/standalone EV charging stations need energy storage. Flywheels and modern flow batteries dominate lead-acid batteries and PHES. Operating a single storage system grows cheaper, but using it for many purposes is tougher. Hybrid energy storage systems are replacing single-type storage systems because they fix their flaws. Swappable battery charging stations are the best way to reduce EV charging time. Manufacturers must standardize energy storage and EV energy management systems to optimize swappable battery storage system benefits. Batteries are good for long-term storage but have environmental issues.

For XFC stations, two distinct distribution strategies are provided. The dc distribution technique offers reduced cost and improved efficiency, whereas the ac distribution method is a mature solution with readily accessible components and well-established standards.

The SST-based DC fast charger offers rectification, voltage step down, and isolation function in a single device, in contrast to the state-of-the-art dc fast chargers that need MV-to LV line-frequency transformers. Compared to the most advanced implementations, the SST-based XFCs are smaller and more efficient, which can lower installation costs by delivering more power on the same station footprint and increase operating profit by minimizing power lost during conversion.

The European Union Member States have established a comprehensive strategy and infrastructure plan to expand electric vehicle charging points gradually. The development of the charging infrastructure within the European Union countries will progress at varying levels due to their significant economic diversity. It is imperative to prioritize placing fast charging points along the TEN-T network and highways.

## Declaration of competing interest

The authors declare that they have no known competing financial interests or personal relationships that could have appeared to influence the work reported in this paper
